# Predicting dynamic expression patterns in budding yeast with a fungal DNA language model

**DOI:** 10.21203/rs.3.rs-7681940/v1

**Published:** 2025-10-14

**Authors:** Kuan-Hao Chao, Majed Mohamed Magzoub, Emily Stoops, Sean Hackett, Johannes Linder, David R. Kelley

**Affiliations:** 1 Department of Computer Science, Johns Hopkins University, Baltimore, MD 21218, USA; 2 Center for Computational Biology, Johns Hopkins University, Baltimore, MD 21218, USA; 3 Calico Life Sciences LLC, South San Francisco, CA 94080, USA

## Abstract

Predicting gene expression from DNA sequence remains challenging due to complex regulatory codes. We introduce a masked DNA language model pretrained on 165 fungal genomes closely related to budding yeast that captures conserved regulatory grammar. Fine-tuning the LM on yeast RNA-seq data—including high-resolution transcriptional regulator induction time courses generated in this study—yielded Shorkie, a model that substantially improves gene expression prediction compared to baselines trained without self-supervision. Shorkie identified canonical transcription factor (TF) binding motifs and tracked their usage across induction experiments. Furthermore, Shorkie accurately predicted variant effects, outperforming leading sequence-to-expression models in *cis*-eQTL classification and achieving high concordance with massively parallel reporter assays. Interpretability analyses revealed Shorkie’s ability to resolve promoter dynamics, splicing signals, and temporal changes in regulatory motif usage. This framework demonstrates that evolutionary-scale pretraining combined with transfer learning substantially improves our ability to decode gene regulation from sequence, providing insights into noncoding variants and regulatory networks.

## Introduction

Predicting gene expression levels from DNA sequence is a fundamental challenge in genomics with broad implications for understanding gene regulation and disease. *Saccharomyces cerevisiae* (budding yeast) has served as the premier model for eukaryotic gene regulation, with ~7,000 genes controlled by hundreds of transcription factors (TFs). Despite decades of work mapping *cis*-regulatory motifs and their regulators^1–11^, quantitative prediction of gene expression from regulatory sequences remains limited. Even sophisticated machine learning models explain at most ~73% of expression variance and rely on hand-crafted rules for motif spacing, orientation, and combinatorial logic^12,13^. This gap highlights the complexity of the regulatory code and motivates new computational approaches.

Supervised deep learning can learn directly from sequence without hand-crafted features^14,15^, but faces a fundamental limitation in yeast: the compact 12 Mb genome provides insufficient training examples, predisposing models to overfitting. Self-supervised DNA language models (LMs) overcome this limitation by learning rich sequence representations from many unlabeled genomes. Models such as DNABERT^16,17^, Evo^18^, and others^19–27^ demonstrate that masked-token prediction captures conserved regulatory syntax and the locations of genes. Parallel advances in protein LMs^28–32^ further validate self-supervised pretraining for extracting functional patterns from sequence.

Despite hundreds of high-quality fungal genomes now available^33^, gene expression data exist for only a handful of species, precluding supervised pan-fungal training. Masked DNA LMs circumvent this limitation: by predicting masked bases, they capture major promoter motifs and the locations of genes without labels. Models pretrained on related species generalize better than those trained on single genomes^17^.

Here, we leverage this paradigm to improve yeast expression modeling. We first pretrained a bidirectional masked LM on diverse fungal genomes using a BERT-style objective. We then fine-tuned this model on high-resolution RNA-seq time courses from transcriptional regulator induction experiments in *S. cerevisiae*^34^, plus publicly available epigenomic and transcriptomic data, creating a high-quality expression predictor called **Shorkie**.

By combining evolutionary pretraining with transfer learning, Shorkie outperforms models trained without self-supervision in predicting expression and temporal dynamics of held-out genes. Furthermore, Shorkie delivers robust variant effect predictions in both *cis*-eQTL classification and massively parallel reporter assays. These findings demonstrate that masked language modeling across diverse fungal genomes, coupled with transfer learning, provides a powerful framework for quantitative gene regulation modeling and noncoding variant interpretation in yeast.

## Results

### Yeast language model design and training across evolutionary divergences

**Figure 1.**
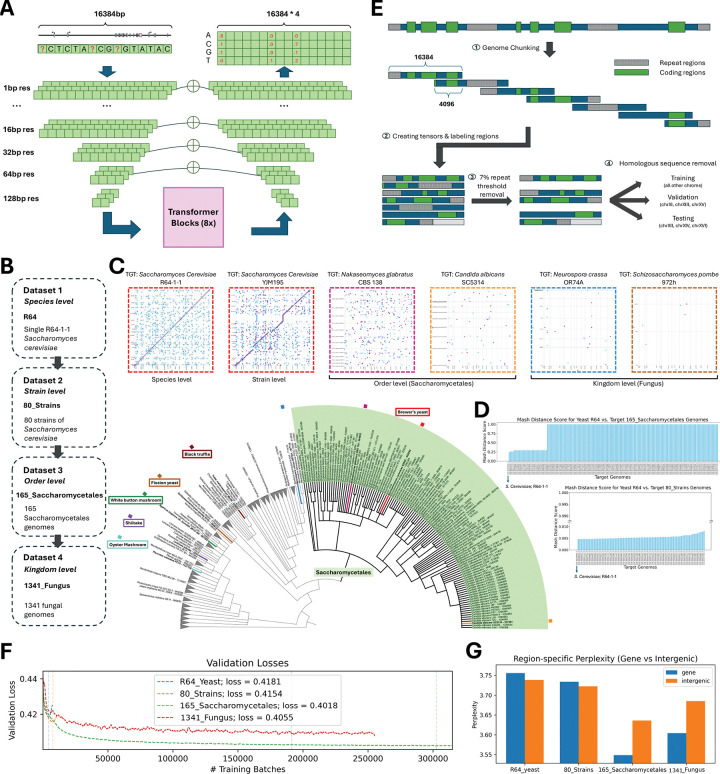
Overview of datasets, preprocessing pipeline, model architecture, and performance metrics for the fungal language model (Shorkie LM). **(A)** Schematic of the Shorkie LM architecture. **(B)** Four datasets employed: single *S. cerevisiae* genome (R64_yeast, species-level), 80 *S. cerevisiae* strains (80_strains, strain-level), 165 Saccharomycetales genomes (165_Saccharomycetales, order-level) with Saccharomycetales highlighted in light green, and 1,341 fungal genomes spanning the kingdom (1341_Fungal, kingdom-level), including common mushrooms such as oyster mushroom (*Pleurotus ostreatus*), shiitake (*Lentinula edodes*), white button mushroom (*Agaricus bisporus*), and black truffle (*Tuber melanosporum*), as well as fission yeast (*Schizosaccharomyces pombe*), and brewer’s yeast (*Saccharomyces cerevisiae*). **(C)** Representative genome distance dot plots for selected genomes from each dataset, with the x-axis representing the R64 *S. cerevisiae* genome and the y-axis representing the comparison genome. **(D)** Mash distance between R64 *S. cerevisiae* genome and genomes in 80_strains and 165_Saccharomycetales. **(E)** Data preprocessing pipeline converting raw genomic data into tensors and labels for Shorkie LM training, validation, and testing. **(F)** Validation loss progression across training steps. **(G)** Comparison of test set perplexity in genic and intergenic regions across four model variants.

We trained a masked DNA LM on more than 1,300 fungal genomes from Ensembl Fungi, which constitutes the same training data as the Species-aware LM developed by Karollus et al. (2024)^22^ (Figure 1A). The model architecture integrates elements from Enformer^35^ and Borzoi^36^, employing a convolutional tower with subsampling followed by eight self-attention blocks operating at 128 bp resolution. We repeated Borzoi’s U-Net upsampling block^37^ seven times to progressive restore single-nucleotide resolution for masked token prediction (detailed model configurations in Figure S1; Methods). This flexible architecture enables fine-tuning at coarser resolutions by removing U-net blocks.

To identify optimal training genomes for *S. cerevisiae*, we prepared four datasets with varying evolutionary divergence (Figure 1B): (1) R64: *S. cerevisiae* reference; (2) 80_strains: 80 *S. cerevisiae* strains; (3) 165_Saccharomycetales: 165 genomes from the Saccharomycetales order; (4) 1341_Fungus: 1,341 fungal kingdom genomes. We inferred phylogenetic relationships with ETE3 using NCBI Taxonomy^38^ and visualized in iTOL^39,40^ (Figure 1B; see Figure S2 for full tree; Methods). We quantified divergence from *S. cerevisiae* R64 using MUMmer dot plots^41^ (Figure 1C) and Mash distances^42^ (Figure 1D). Closely related strains (e.g., YJM195 at Mash ≈ 0.01) exhibited near-continuous synteny, whereas taxa such as *C. albicans* and *N. glabratus* showed fragmentation (Mash 0.25–1). Distant outgroups (*S. pombe*, *N. crassa*) yielded negligible alignments (Figure 1C).

We prepared genomes for LM training by masking repetitive elements^43–48^ (Figure S3A–B; Methods), segmenting into overlapping 16,384 bp windows with 4,096 bp stride, and excluding windows with >7% repetitive content. We assessed gene count per window (Figure S4A–B), coding-to-noncoding ratios per window (Figure S4C–D), repetitive region distribution (Figure S4E–F), and gene annotation completeness (Figure S4G–I; Methods). To focus learning on regulatory sequences, we down-weighted the loss function by 0.1 at coding (72% of *S. cerevisiae* R64) and repetitive (7.39%) positions (Figure S3C; Methods). Training/validation/test sets were split by *S. cerevisiae* chromosomes. Validation/test sets included only *S. cerevisiae*; the training set included all genomes after removing sequences homologous to validation or test sets using minimap2 at 20% divergence cutoff^49,50^ (Figure 1E; Figure S3D–E; Methods).

Evolutionary divergence correlated with training complexity, reflected in higher training loss. The 165_Saccharomycetales model achieved the lowest validation loss, outperforming the more divergent 1341_Fungus and avoiding the overfitting observed with 80_strains and R64 (Figure 1F). This optimal performance extended to the test set (Figure 1G) and held across alternative architectures (two residual CNN baselines and a larger U-Net-transformer) (Figure S5B–D, Figure S6). The 165_Saccharomycetales-trained model, hereafter “Shorkie LM”, thus represents the optimal evolutionary scale for *S. cerevisiae* generalization.

### Shorkie LM captures regulatory conservation and generalizes across diverse fungi

**Figure 2.**
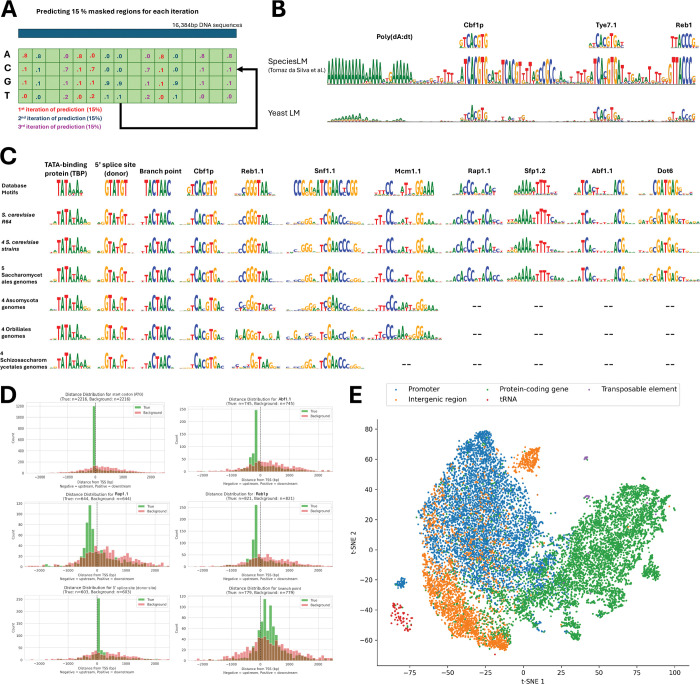
Shorkie LM identifies conserved transcription factor binding motifs across fungal genomes. **(A)** Position probability matrix (PPM) reconstruction from DNA sequences using the fungal language model Shorkie LM. **(B)** Comparative analysis of SMT3 promoter predictions (chrIV:1,469,090–1,469,198) by Shorkie LM and the Species-aware DNA LM^22*,*52^, highlighting key motifs including poly(dA:dT), Cbf1, Tye7, and Reb1. **(C)** Summary of known motifs detected by Shorkie LM across six datasets: (1) reference *S. cerevisiae* genome; (2) four randomly selected *S. cerevisiae* strains; (3) five genomes from the Saccharomycetales order; (4) four genomes from the Ascomycota phylum; (5) four genomes from the Orbiliales order; and (6) four genomes from the Schizosaccharomycetales order. TF-MoDISco-identified motifs include TATA-binding protein, 5′ splice site (donor), branch point, Cbf1p, Reb1.1, Snf1.1, Mcm1.1, Rap1.1, Sfp1.2, Abf1.1 and Dot6. **(D)** Histograms depicting enrichment of TF-MoDISco-identified motifs upstream of transcription start sites (TSS) relative to background distributions in *S. cerevisiae*, and enrichment of 5’ splice sites (donors) and branch points within genic regions. **(E)** t-SNE embeddings of different genomic elements from the first self-attention layer of Shorkie LM.

Transcription factor (TF) binding motifs are fundamental regulatory units^51^, and previous studies demonstrate that masked DNA LMs learn subtle motif co-occurrence patterns^22^. We evaluated Shorkie LM’s motif identification by segmenting the *S. cerevisiae* genome (Figure 1B), randomly masking 15% of bases, and iteratively imputing them to reconstruct position probability matrices (Figure 2A; Methods). In the SMT3 promoter, Shorkie LM identified canonical motifs including Poly(dA:dT), Cbf1, Tye7, and Reb1, consistent with prior analyses^52^ (Figure 2B). This alignment-free approach enables flexible sequence probability derivation^19^.

We assessed Shorkie LM across six fungal datasets spanning different evolutionary distances (see Methods). Following prediction, we employed TF-MoDISco-lite^53,54^ for *de novo* motif clustering and matched clusters to yeast motif databases^55–60^ (Methods). Motif conservation varied across evolutionary distance. Shorkie recovered core regulatory motifs and features, including TATA-binding protein (TBP)/TATA elements, start codons, splice sites, and TF binding sites such as Cbf1, Reb1, and Snf1. Mcm1.1 was absent in Schizosaccharomycetales, which lack a direct homolog; this role is instead served by the functionally analogous MADS-box transcription factor Map1^61–64^. Motif conservation declined beyond the Saccharomycetales order, consistent with model training metrics (Figure 1G; Figure 2C). See Supplemental Figures S7 and S8 for comprehensive motif discovery results.

To validate biological relevance, we mapped TF-MoDISco-derived motifs onto the *S. cerevisiae* genome, assigning motifs to their nearest genes and computing transcription start site (TSS) distances. Compared with random controls, TBP, Cbf1p, Reb1.1, Mcm1.1, and Snf1.1 showed promoter enrichment (Figure 2D; Figure S7). The 5’ splice donor site localized downstream of TSSs, while branch points distributed broadly within genes. Five randomly selected Saccharomycetales genomes produced similar enrichments (Figure S8). Additionally, Shorkie LM’s first attention layer effectively differentiated genomic features by embedding patterns (Figure 2E), as did subsequent layers (Figure S9).

These results demonstrate that Shorkie LM captures conserved regulatory grammar, accurately identifies motifs, and generalizes across substantial evolutionary distances.

### Shorkie: LM transfer learning enables improved gene expression prediction

**Figure 3.**
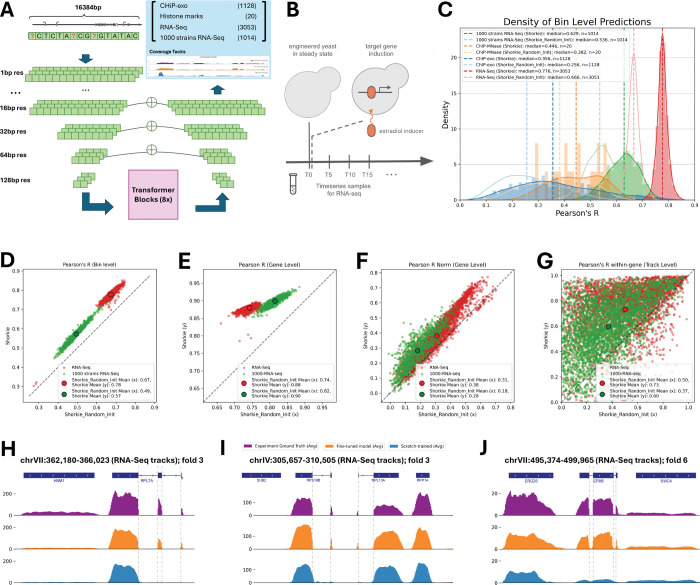
Shorkie architecture and RNA-seq prediction performance across multiple scales. **(A)** Shorkie architecture: U-Net model with eight transformer blocks. All layers inherit pretrained Shorkie LM weights; task-specific output heads (blue) predict perturbation timepoint RNA-seq (n = 3,053), 1000-strain RNA-seq (n = 1,014), ChIP-exo (n = 1,128) and ChIP-MNase histone marks (n = 20). **(B)** Yeast cells were grown to steady state, as determined by culture density, prior to addition of b-estradiol to the culture and subsequent sampling. **(C)** Distribution of bin-level Pearson’s R on held-out test data for each track type, comparing Shorkie and Shorkie_Random_Init. **(D-G)** Scatter plots comparing Shorkie and Shorkie_Random_Init for RNA-seq tracks at **(D)** bin-level Pearson’s R; **(E)** gene-level Pearson’s R; **(F)** quantile-normalized and mean-centered gene-level Pearson’s R; **(G)** gene-by-gene, track-level Pearson’s R. **(H-J)** RNA-seq coverage snapshots of *S. cerevisiae* test set gene loci: **(H)** chrVII:362,180–366,023 (RPL7A); **(I)** chrIV:305,657*–*310,505 (RPS16B and RPL13A); and **(J)** chrVII:495,374–499,965 (EFM5).

Building on the strong self-supervised sequence foundation, we developed Shorkie, a supervised model predicting RNA-seq and ChIP-exo/MNase aligned coverage tracks at 16 bp resolution from DNA sequences. Starting with the LM architecture, we removed its final four upsampling layers and added task-specific output heads (Figure 3A; complete configurations in Figure S10; Methods). We curated 2,162 experimental tracks for training: 1,128 ChIP-exo^65^, 20 ChIP-MNase^65^, and 1,014 RNA-seq datasets from various yeast isolates^66^.

In addition, we generated 3,053 new high-resolution induction RNA-seq timepoints using a protocol adapted from Hackett et al.^34^ (Figure 3B), bringing the total to 5,215 experimental tracks. Key protocols and description of chemostats (ministat array), culture conditions, library preparation, data pre-processing and quality controls are detailed in Methods (Figure S11–S12).

To evaluate the impact of pretraining, we compared transfer learning from Shorkie LM against random initialization (Shorkie_Random_Init) across eight-fold cross-validation (Figure S13). On the transcriptional regulator induction RNA-seq test data, Shorkie achieved median bin-level Pearson’s R of 0.78 versus 0.67 for Shorkie_Random_Init, shifting the correlation distribution upward (Figure 3C) and boosting per-track correlations (Figure 3D).

Gene-level aggregation across exon-overlapping bins (Methods) yielded mean Pearson’s R of 0.88 for Shorkie versus 0.74 for Shorkie_Random_Init (Figure 3E). Normalized gene-level correlations (quantile-normalized per experiment and mean-centered per gene) confirmed this advantage (Figure 3F; Methods). After averaging track-specific performance across tracks for each gene, Shorkie exceeded Shorkie_Random_Init in 87.8% of genes, particularly at higher expression levels (Figure 3G; Figure S14L; Methods).

To understand the model’s regulatory focus, we analyzed self-attention weights centered on the three-exon gene EFM5 and housekeeping gene RPL7A (Methods). Attention from both the pretrained LM and Shorkie highlighted genic and discrete intergenic regulatory regions (putative promoters), whereas Shorkie_Random_Init produced diffuse attention patterns (Figure S15). Test loci visualization confirmed Shorkie’s accurate prediction of intronic coverage drops and expression profiles, contrasting Shorkie_Random_Init less precise predictions (Figure 3H–J).

These results demonstrate that supervised transfer learning from pan-fungal self-supervised pretraining provides robust, generalizable representations of exon-intron structure and regulatory grammar, yielding substantial improvements in expression prediction.

### Shorkie transfer learning preserves regulatory motif recognition

To identify sequence patterns utilized by Shorkie, we performed *in silico* saturation mutagenesis (ISM) on 500 nt promoter windows (−450 to +50 relative to TSS) for three gene cohorts: 137 ribosomal protein (RP), 64 ribosome and rRNA biosynthesis (RRB), and 3,258 additional protein-coding genes. For Shorkie and Shorkie_Random_Init, we computed ISM importance maps by averaging predictions across that model’s eight cross-validation folds. We then compared these ISM maps to per-base information content derived from the pretrained Shorkie LM’s predictive probability distribution relative to the genomic background.

**Figure 4.**
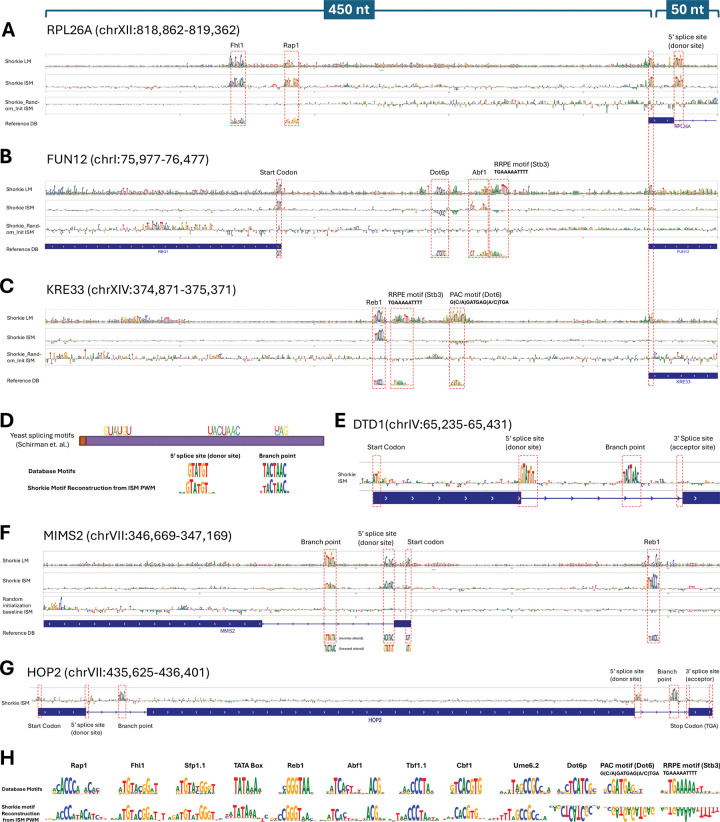
Shorkie uses promoter and splicing motifs learned during pretraining. **(A-C)** Promoter regions (−450 to +50 bp relative to the TSS; 500 bp total) of RPL26A (chrXII:818,862–819,362), FUN12 (chrI:75,977–76,477), and KRE33 (chrXIV:374,871–375,371). Rows 1–3 show DNA logos from Shorkie LM, and ISM maps from Shorkie (fine-tuned) and Shorkie_Random_Init (no self-supervision pretraining). Row 4 shows gene annotations from IGV JS^80^. The Shorkie LM PWMs were generated from PPMs (Figure 2A; Methods), whereas the Shorkie and Shorkie_Random_Init ISM maps were produced via an ISM analysis that systematically substituted each nucleotide with the three alternatives. **(D)** Canonical S. cerevisiae splicing motifs^81^. **(E-G)** Shorkie ISM maps for splicing motifs in DTD1, MIMS2, and two-intron gene HOP2. **(H)** TF-MoDISco-identified motifs on Shorkie ISM maps: curated yeast database motifs (top) and Shorkie-derived motifs (bottom).

In RP promoters like RPL26A (Figure 4A, Figure S16), both Shorkie LM and Shorkie recovered the fork-head-binding IFHL motif, typically located ~50–80 bp upstream of the TSS. This motif is recognized by the winged-helix domain of Fhl1, which upon phosphorylation recruits the coactivator Ifh1, linking TOR/PKA signaling to transcriptional activation^67–69^. Both models also identified the UASrpg element (Upstream Activation Sequence, ribosomal protein genes), bound by the pioneer factor Rap1, which recruits chromatin remodelers, scaffolds Fhl1–Ifh1 complexes, displaces the +1 nucleosome, and establishes nucleosome-depleted regions essential for preinitiation complex assembly^70–73^.

In RRB promoters, such as FUN12 (Figure 4B) and KRE33 (Figure 4C), Shorkie LM captured the RRPE motif (5’-TGAAAAATTTT-3’), bound by the repressor Stb3, and the PAC motif (5’-GCGATGAGATGAG-3’), recognized by Dot6/Tod6 repressors. These *cis*-elements coordinate rRNA processing and ribosome assembly genes during the cell cycle and stress responses^74–79^. Shorkie recapitulated RRPE and PAC motifs and additionally detected Abf1 and Reb1 binding motifs. See Figure S17 for four additional protein-coding genes.

Within genes, Shorkie LM and Shorkie detected canonical splicing signals (Figure 4D)^81^: in DTD1 (Figure 4E), MMS2 (Figure 4F), and the multi-exon HOP2 (Figure 4G), Shorkie ISM maps delineated the 5’ splice donor and branch-point^81–84^. Models were insensitive to acceptor-site mutations—an observation mirrored by Tomaz da Silva et al.^52^, which likewise fails to reconstruct 3’ acceptor motifs. TF-MoDISco analysis recovered additional motifs including TATA, Sfp1, Tbf1, Cbf1, and Ume6 (Figure 4H).

Across all ISM analyses, Shorkie’s ISM maps preserved regulatory motif signatures acquired during language model pretraining, whereas Shorkie_Random_Init failed to recover key motifs. Thus, Shorkie leverages learned regulatory and genic features to improve predictions.

### Shorkie captures dynamic *cis*-regulatory motif changes across time-course TF inductions

Building on Shorkie’s ability to identify static cis-regulatory elements, we investigated temporal motif usage during TF induction. We performed ISM across promoters (−450 to +50 nt relative to TSSs) of Saccharomyces Genome Database (SGD)-curated TF targets^85^ to generate time-resolved maps (Methods).

**Figure 5.**
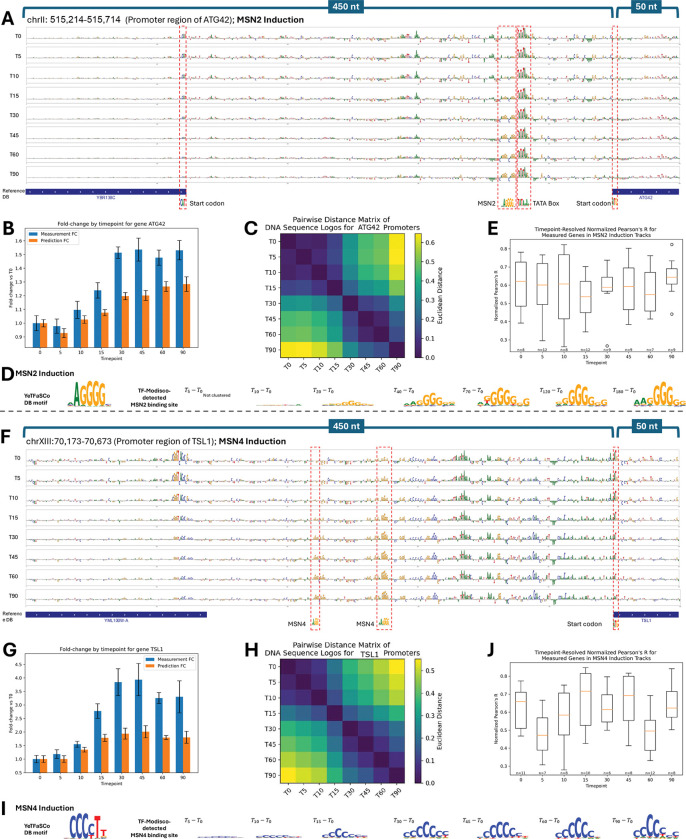
Time-course analysis of stress-responsive transcription factor induction. **(A-E)** MSN2 induction at the ATG42 promoter region (−450 to +50 bp relative to the TSS; chrII:515,214–515,714), sampled at seven timepoints labeled in minutes. **(A)** Shorkie ISM sequence logos: rows correspond to successive timepoints (top to bottom), with the bottom row showing the reference. Key TF-binding motifs are annotated. **(B)** Experimental fold-change in reads per million (RPM) (blue) versus Shorkie-predicted signal (orange) across the ATG42 locus at each timepoint. **(C)** Heatmap of pairwise Euclidean distances between ISM logos, illustrating temporal divergence in motif strength and composition. **(D)** TF-MoDISco-identified motifs extracted from ΔT ISM matrices relative to $T_{0}$. **(E)** Boxplot of normalized Pearson’s R between experimental and predicted profiles across all *S. cerevisiae* genes for MSN2 induction at each timepoint. **(F–J)** MSN4 induction at the TSL1 promoter region (−450 to +50 bp relative to the TSS; chrXIII:70,173–70,673), with panels analogous to **(A–E)**.

We first examined MSN2, a C2H2 zinc-finger TF that activates ~200 stress-responsive genes via STRE motifs^86–89^. Shorkie’s ISM maps at the ATG42 promoter revealed progressive STRE sharpening over 0–90 min (Figure 5A), mirroring RNA-seq fold-changes (Figure 5B; Methods). Euclidean distance heatmaps quantified temporal ISM divergence (Figure 5C), with normalized, mean-centered Pearson’s R between experimental and predicted RNA-seq across MSN2 perturbations ranging from 0.55 to 0.65 (Figure 5D). TF-MoDISco analysis of ΔT ISM maps captured average motif kinetics (Figure 5E; Figure S18A–D show another example at the GLK1 promoter; Methods).

We next examined MSN4, an MSN2 paralog rapidly induced upon stress^90^. Shorkie’s ISM analysis at the TSL1 promoter showed similar STRE dynamics, corresponding with RNA-seq fold-changes (Figure 5F–G) and reflected in Euclidean distance heatmaps (Figure 5H). TF-MoDISco analysis of ΔT ISM maps quantified temporal motif changes, with normalized Pearson’s R between experimental and predicted RNA-seq ranging 0.45–0.70 (Figure 5I–J; Figure S18E–H, AYR1 promoter).

Finally, we investigated MET4, a bZIP co-activator recruited to E-box motifs (TCACGTG) by cofactors Cbf1 and Met31/Met32^91,92^. Shorkie’s E-box ISM maps inversely correlated with TF induction and were attenuated by cofactor binding, suggesting capture of cofactor-mediated recruitment rather than direct MET4–DNA binding (Figure S19; see Discussion).

These results demonstrate that Shorkie dynamically tracks cis-regulatory motif usage across TF induction time courses, recapitulating activation kinetics and providing insights into temporal regulatory grammar.

### Shorkie predicts promoter variant effects validated by MPRAs

Massively parallel reporter assays (MPRAs) provide high-throughput measurements of *cis*-regulatory activity, enabling regulatory syntax hypothesis testing and variant interpretation. While MPRA data are not ideal for Shorkie due to its training on large endogenous sequences rather than short reporter constructs, we still expect reasonable concordance predicting MPRA sequences after marginalizing surrounding context.

We assessed Shorkie using the Random Promoter DREAM Challenge MPRA dataset^93^ containing a held-out set of 71,103 sequences across eight categories: native yeast promoters; random 80-bp oligonucleotides; high-expression sequences; low-expression sequences; sequences challenging prior models; single-nucleotide variant (SNV) perturbations; motif perturbations; and motif-tiling constructs. Each sequence was assayed in ~100 cells for precise expression estimates^93^. To make marginal predictions with Shorkie, we replaced MPRA constructs upstream of TSS for selected “background” genes and averaged predictions across backgrounds.

**Figure 6.**
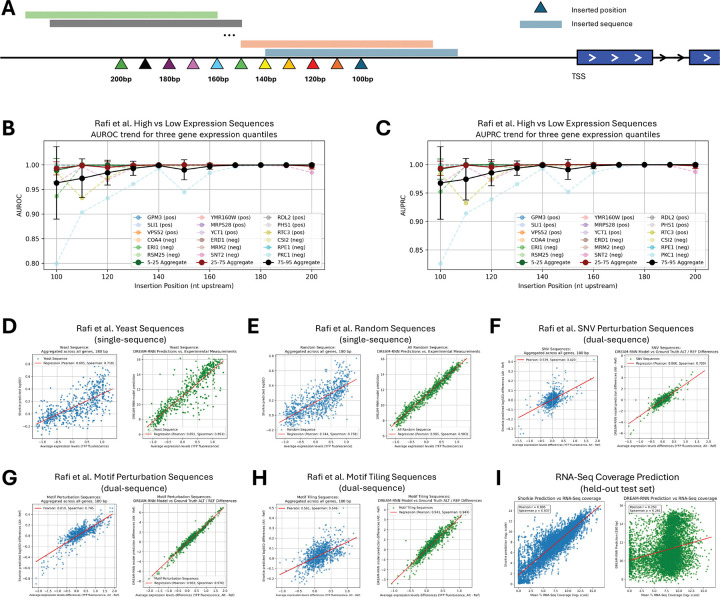
Evaluation of Shorkie’s predictions of promoter variant effects using MPRA data. **(A)** Experimental schematic showing MPRA sequences inserted at positions 100–200 bp upstream of the TSS in 10 bp increments for selected yeast genes. **(B-C)** Classification performance distinguishing high- versus low-expression sequences across upstream insertion sites assessed by **(B)** AUROC and **(C)** AUPRC. Genes were stratified into three RNA-seq expression quantiles (5–25%, 25–75%, and 75–95%); dashed colored lines represent individual genes, and black lines depict mean ± standard error. **(D–E)** Comparison between Shorkie predictions (log fold-change scores) and DREAM-RNN model predictions with experimentally measured expression for **(D)** native yeast sequences and **(E)** challenging sequences. **(F–H)** Model performance evaluated for specific regulatory variant sets: **(F)** single-nucleotide variants (SNV), **(G)** motif perturbations, and **(H)** motif tiling constructs. **(I)** RNA-seq coverage predictions comparing Shorkie and DREAM-RNN against experimentally measured coverage.

To characterize positional effects, we selected 10 forward-strand and 12 reverse-strand genes representing low (5–25th percentile), medium (25–75th percentile), and high (75–95th percentile) pre-induction RNA-seq expression quantiles. We systematically inserted MPRA sequences at eleven positions, stepping every 10 bp from 200 to 100 bp upstream of the TSS, and quantified regulatory impact as the log fold-change in downstream gene expression (Figure 6A; Methods).

Across tested genes, high-expression sequences yielded positive log fold-change scores, while low-expression sequences typically produced negative scores (Figure S21A–B). Effect magnitudes modestly increased with greater TSS distance. Framing high- versus low-expression sequence prediction as binary classification, Shorkie achieved near-perfect discrimination at each insertion site (AUROC and AUPRC >0.95; Figure 6B–C; Figure S21C–D). The position 180 bp upstream was selected for subsequent analyses (Figure S20A).

Shorkie’s marginalized predictions strongly correlated with experimental MPRA measurements: Pearson’s R of 0.70 for native yeast promoters, 0.74 for random sequences, and 0.70 for challenging sequences (Figure 6D–E, Figure S20B–D). For variant-specific categories, comparing reference-alternate differences, correlations were 0.54 for SNV perturbations, 0.82 for motif perturbations, and 0.56 for motif-tiling constructs (Figure 6F–H). While the best-performing MPRA-trained model, DREAM-RNN outperformed Shorkie on MPRA predictions, Shorkie’s concordance is assuring given zero-shot generalization from endogenous genome training. Moreover, Shorkie outperformed DREAM models when predicting endogenous gene expression, highlighting that context drives performance differences (Figure 6I). In sum, these results demonstrate Shorkie’s robust capability to predict promoter-driven expression.

### Shorkie predicts *cis*-eQTL regulatory impacts

**Figure 7.**
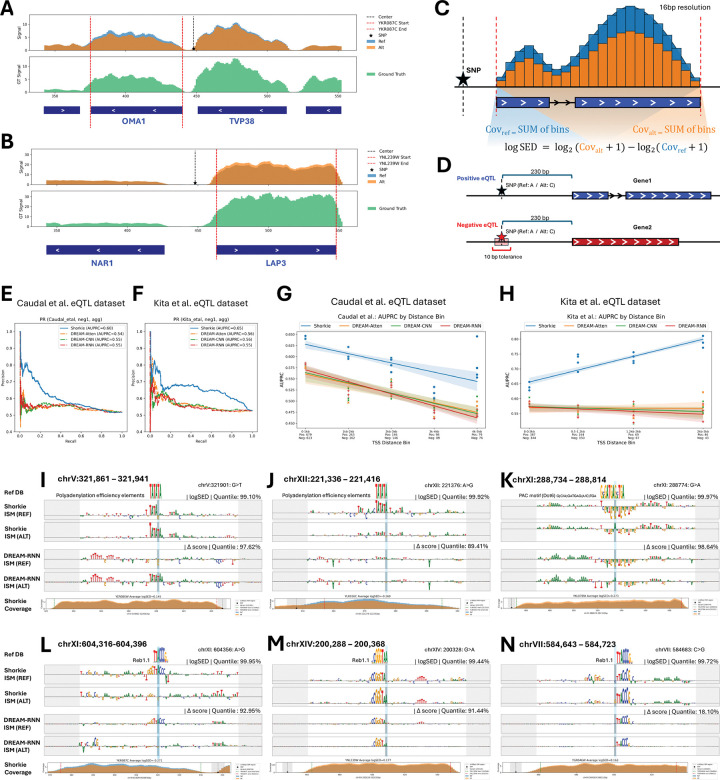
Shorkie accurately predicts cis-eQTL variant effects. **(A)** Positive eQTL example demonstrating reduced expression associated with the alternate allele at OMA1 (chrXI:603,195–604,232). **(B)** Positive eQTL showing increased expression associated with the alternate allele at LAP3 (chrXIV:200,569–201,933). **(C)** Computation of log fold-change expression scores for evaluating variant effects. **(D)** Generation of negative eQTL controls matched by genomic characteristics from ~1,000 natural yeast isolates. **(E,F)** Precision-recall (PR) curves comparing Shorkie and DREAM models for Caudal et al. **(E)** and Kita et al. **(F)** datasets. **(G,H)** AUPRC scores by TSS distance bins in Caudal et al. **(G)** and Kita et al. **(H)** datasets. **(I–N)** ISM maps centered on eQTL SNPs, highlighting regulatory motifs identified by Shorkie (SNP in light-blue) and DREAM-RNN, with adaptor sequences in gray.

Predicting how regulatory variants alter gene expression is critical for dissecting genetic association mechanisms. We used Shorkie to interpret yeast *cis*-eQTL effects^66,94,95^. For instance, at the OMA1 locus, the eQTL alternate allele reduced Shorkie’s predicted RNA-seq coverage relative to reference (Figure 7A)^66,95^, while at LAP3, the lead eQTL alternate allele increased predicted coverage (Figure 7B). We quantified variant effects via gene expression log fold-change (Figure 7C; Methods).

We benchmarked Shorkie on eQTL datasets from Caudal et al. (1,901 local *cis*-eQTLs from ~1,000 yeast isolates; Figure S22A–B; Methods)^66,95^ and Kita et al. (683 eQTLs categorized as “Promoter,” “UTR5,” “UTR3,” and “ORF”; Methods)^94^. Negative controls were generated by randomly selecting noncoding SNPs matched by reference/alternate alleles, TSS distance, and minor allele frequency ≥5% (Figure 7D; Figure S22C;Figure S23A; Methods).

Compared to top DREAM Challenge models (DREAM-CNN, DREAM-RNN, DREAM-Atten)^93^, Shorkie achieved superior ROC and PR metrics for both Caudal et al. (Figure 7E; Figure S22E) and Kita et al. datasets (Figure 7F; Figure S23), with higher AUROC and AUPRC scores across all TSS distance bins (Figure 7G–H).

ISM analyses of eQTL SNP loci revealed allele-specific remodeling of key *cis*-regulatory elements: creation (Figure 7I) or loss (Figure 7J) of polyadenylation efficiency motifs; disruption of PAC motif bound by Dot6/Tod6 repressors, correlating with increased expression (Figure 7K); and Reb1 motif alterations either weakening binding (Figure 7L) or strengthening it (Figure 7M–N). See Figure S24 for additional examples.

## Discussion

In this study, we demonstrate that pretraining a language model on Saccharomycetales order genomes strikes an optimal balance between dataset size and diversity with regulatory conservation for learning the *S. cerevisiae cis*-regulatory grammar. Integrating convolutions, transformers, and residual connections within a U-Net architecture, Shorkie LM attains low perplexity on held-out *S. cerevisiae* chromosomes and reconstructs canonical TF binding motifs *de novo* across diverse fungal species. Transfer learning from this foundation into Shorkie substantially improves RNA-seq and ChIP-exo/MNase coverage prediction, raising median bin-level Pearson’s R from 0.67 (random initialization baseline) to 0.78 and gene-level Pearson’s R from 0.74 to 0.88.

TF-MoDISco and ISM analyses show that Shorkie captures both static and dynamic motif signatures. The model recovers static motifs including Poly(dA:dT) tracts, Cbf1, Reb1 and canonical splice-site signals, confirming that evolutionary pretraining internalizes core regulatory elements. During transcription factor induction time courses, Shorkie dynamically tracks regulatory changes: STRE motif signals sharpen during MSN2 and MSN4 responses, and E-box engagement reflects cofactor-mediated MET4 activation.

While ISM and TF-MoDISco correctly identify E-box motifs at MET4 target promoters, they also highlight secondary signals with unclear mechanisms. These features may reflect persistent SCF^Met30^-mediated ubiquitination of Met4^96^, transcriptional squelching via Mediator/SAGA cofactor sequestration^97,98^ or feedback from intracellular sulfur metabolites^99^. Disentangling these regulatory layers demands targeted experiments—e.g. Met4 and cofactor ChIP-seq, ubiquitination-deficient Met4 variants and time-resolved metabolomics. Such studies could distinguish direct DNA binding from indirect regulatory cascades and guide future Shorkie applications to transcriptional networks.

Shorkie’s ability to learn regulatory patterns from perturbation experiments opens new avenues for network inference. Genome-wide perturbation screens could systematically map regulator-target relationships through sequence interpretation, while time-series experiments could reveal kinetic parameters governing transcription, mRNA degradation, and splicing. These applications extend Shorkie beyond prediction toward mechanistic discovery. Current limitations point to specific technical challenges. ChIP-exo was the most challenging assay: predictions track broad trends but systematically underestimate extreme, narrow peaks. This reflects the zero-inflated, heavy-tailed nature of ChIP-exo data and suggests that variance-stabilizing target transforms (e.g., square-root) or distribution-aware losses (e.g., focal loss) could improve performance (Figure S25). Similarly, Shorkie RNA-seq time course predictions show compressed dynamic range relative to experimental measurements (Figure 5B,G).

Yeast exemplifies where self-supervised pretraining excels. With a ~12 Mb compact genome, *S. cerevisiae* cannot support large-scale supervised learning from scratch. MPRA approaches offer synthetic training data but sacrifice native genome context, creating a fundamental tradeoff between scale and biological authenticity. Our evolutionary pretraining strategy complements these efforts, allowing Shorkie to learn representations grounded in native promoters. Future work may explore clever combinations to exploit the strengths of the different approaches.

We propose that genome size and label abundance jointly determine when pretraining yields benefit. Small-genome organisms with limited experimental data (like yeast) benefit most from phylogenetically informed pretraining. Large-genome species (like mammals) with extensive experimental resources may show smaller pretraining gains, though benefits could persist in data-sparse contexts and cross-species transfer. Systematic studies varying taxonomic scope, phylogenetic distance, and experimental data volume will define the boundaries of this pretraining regime.

Our results establish masked nucleotide pretraining as a powerful foundation for regulatory genomics. By training at optimal evolutionary scales, models learn transferable representations that substantially improve sequence-to-expression prediction. While technical challenges remain, this framework provides a scalable path toward mechanistic understanding of gene regulation across diverse biological systems.

## Online Methods

### Fungal genome and annotation download, filtering, and biotype partitioning

We downloaded the main species table containing accession IDs, species names, and assembly and database identifiers from Ensembl Fungi release 59 (https://ftp.ebi.ac.uk/ensemblgenomes/pub/release-59/fungi/species_EnsemblFungi.txt) and used to retrieve all genomic and annotation data. For each taxon, we retrieved the unmasked genome FASTA file and its XML metadata, plus the corresponding GTF annotation file.

We filtered each genome FASTA was filtered by parsing its XML to determine assembly level (“chromosome” vs. “scaffold”). We retained both chromosome- and scaffold-level assemblies, discarding contigs shorter than 32,768 bp. We renamed remaining contigs according to a unified convention (chrI through chrXVI for yeast, original names otherwise). We produced a cleaned species manifest listing assembly levels, chromosome counts, and total base counts for all retained genomes. We partitioned each annotation file into subsets based on gene biotype (e.g. protein-coding, non-coding, rRNA, etc.).

### Shorkie LM model architecture

Shorkie is a U-Net transformer-based model that processes 16,384-bp genomic windows and outputs a probability distribution over the four nucleotides (A, C, G, T) at each position (Figure S1). It contains 13,665,828 parameters (13,651,812 trainable; 14,016 non-trainable).

### Encoder (down-sampling) path

The encoder begins with a 1D convolution (kernel size = 11, filters = 96), projecting the input tensor to a feature map of shape (16,384 × 96). Seven successive residual down-sampling stages follow. At stage i, the feature map is passed through a residual convolutional block: batch normalization → Gaussian Error Linear Unit (GELU) activation^100^ → Conv1D (kernel size = 5; filters = $C_{i}$) → dropout (p = 0.05) → learned residual scaling. The block output is added to its input, and a MaxPooling1D layer (pool size = 2) reduces the sequence length in half. Channel widths increase across stages as $C_{i}\in [96,\;128,\;160,\;192,\;256,\;320,\;384]$, while the sequence length decreases by 2 × each stage from 16,384 (1-bp) to 128 (128-bp).

### Transformer bottleneck

At 128 bp resolution, the (128 × 384) feature map was feed into a stack of eight Transformer blocks to capture long-range dependencies. Each layer uses LayerNorm, then multi-head self-attention (model dim = 384; 8 heads; key dim = 64) with residual dropout (p = 0.05), followed by a two-layer position-wise feedforward network (Dense → Dropout → ReLU → Dense → Dropout) with a residual connection. This bottleneck integrates context across the full input span.

### Decoder (up-sampling) path and output

The decoder mirrors the encoder in seven up-sampling stages. Each stage applies BatchNorm and GELU, a channel-preserving projection (Dense 384→384), and UpSampling1D (size = 2) to double the sequence length (e.g., 128 → 256 → … → 16,384). The up-sampled features are merged with the corresponding encoder features via U-Net–style skip connections to restore fine-grained detail, then refined by a depthwise-separable convolution (kernel size = 3; filters = 384), BatchNorm, and GELU. A final 1×1 Conv1D (filters = 4) followed by softmax yields a per-position probability distribution over {A, C, G, T}. (Nearest-neighbor upsampling follows the UpSampling1D definition.)

### Phylogenetic tree reconstruction and fungal genomes distance estimation

#### Phylogenetic tree creation

We converted all species names to NCBI Taxonomy identifiers (TaxIDs) with the ETE Toolkit Python API^38^. To reconstruct a minimal spanning tree that includes only our taxa of interest, we ran the ete-ncbiquery command-line module with our TaxID list and requested Newick-formatted output. This step pruned all non-target lineages while preserving branch lengths and hierarchical relationships. We loaded the resulting Newick file into ETE to verify correct monophyletic groupings and confirm presence of every target species in NCBI. For visualization, we uploaded the pruned tree to the Interactive Tree Of Life (iTOL v5) web server^39,40^ and overlaid custom annotations (e.g., colored clade highlights) using iTOL dataset templates (Figure S2).

#### Pairwise genomic distance estimation using alignment- and sketching-based methods

We quantified genomic divergence of the 1,341 fungal assemblies relative to the *S. cerevisiae* R64 reference using complementary alignment-based and sketching-based methods. For the alignment-based approach, we ran MUMmer4’s nucmer^41,101^ with 40 CPU threads to align each cleaned FASTA against the R64 reference, producing a delta file of all maximal unique matches. We then extracted detailed alignment statistics with show-coords -lcr, yielding tables of reference and query start-end positions, block lengths, percent identity, and coverage for every alignment block. To visualize large-scale synteny and structural variation, we generated dot plots for each genome pair using MUMmerplot. These plots enable rapid inspection of collinearity breaks, inversions, and translocations across the fungal assemblies.

For rapid, alignment-free genomic distance estimation, we applied two sketching-based tools: Dashing2^102^ and Mash^42^ on the same genome pairs. Dashing 2 leverages the SetSketch data structure^103^ (using a truncated logarithm of hashed k-mers) together with ProbMinHash^104^ for multiplicity-aware sketching. Mash^42^ employs classical MinHash^105^ on each genome’s k-mer set (default k = 21, sketch size s = 1,000) to estimate Jaccard similarity^106^.

### Shorkie LM data preprocessing

#### Repetitive region detection and masking

Most fungal assemblies in the Ensembl database lack adequate soft-masking of repetitive elements. To address this, we retrieved 1,501 genomes from the Ensembl FTP site and implemented a two-tiered repeatmasking pipeline. First, we generated a *de novo* repeat library for each genome using RepeatModeler v2.0^44^, which integrates multiple discovery algorithms, RepeatScout, RECON, and LTR_retriever^44,47,48^, to capture both interspersed and structural elements unique to each assembly, and then merged the resulting consensus sequences with curated entries from Dfam^46,107^. Next, we ran RepeatMasker against this custom library (plus standard repeat databases), employing RMBlast, a RepeatMasker compatible version of the standard NCBI blastn program (https://www.repeatmasker.org/rmblast/), for sensitive and high-throughput alignments. We enabled the “-xsmall“ option to soft-mask repeats in lowercase thereby preserving original sequence length and coordinates and the “-gff“ flag to output annotations in GFF3 format. Finally, we applied the DUST algorithm (https://meme-suite.org/meme/doc/dust.html) via the MEME suite^43^ (default cutoff score threshold 20) to soft-mask residual low-complexity regions (Figure S3A). After the pipeline, 1,341 genomes were successfully masked and used for further preprocessing. We validated the workflow by comparing Ensembl’s original soft-masked regions with our custom masks in six representative assemblies and observed strong concordance (Figure S3B). We then partitioned each genome into 16,384-bp windows with a 4,096-bp stride and applied a 7% repeat-content threshold for quality control, excluding ~20% of windows from the training, validation, and test sets (Figure S3C).

#### Homologous and paralogous sequence removal between training, validation and test sets

To guard against data leakage and ensure truly independent evaluation splits, we developed a three-step homology-filtering pipeline that removes any training sequences sharing appreciable similarity with those in our validation or test sets^50^. First, we aligned every training sequence against both the validation and test sets using Minimap2 (v2.28-r1209; assembly-to-assembly mode, “-x asm“)^49^ to produce PAF files that report, for each query, the coordinates and match statistics of all detected alignments. From each PAF file, we extracted two metrics for every query-target pair:

- Coverage = matching bases (PAF field 10) / query length (PAF field 2)
- Identity = matching bases (PAF field 10) / alignment block length (PAF field 11)

These ratios quantify how much of a training sequence overlaps with other splits and how similar those overlaps are. Finally, we removed any training sequence for which both coverage ≥ 5 % and identity ≥ 30 % in any alignment, yielding a leakage-free training corpus (Figure S3D). The alignment scatter plots for train-test and train-validation splits for each dataset are shown in Figure S3E.

#### Evaluating fungal genome annotation completeness using BUSCO

We first retrieved and unpacked the BUSCO v5 fungal lineage dataset (fungi_odb10, 758 orthologs; 2024–01-08; https://busco-data.ezlab.org/v5/data/lineages/fungi_odb10.2024-01-08.tar.gz) to establish a consistent benchmark. For each of the 1,341 assemblies, we extracted all proteins from the Ensembl Fungi release 59 GFF annotation and its corresponding genome using gffread^108^. We then ran BUSCO^109^ in protein mode (-m proteins) on each proteome. BUSCO classifies each ortholog as “Complete (single-copy or duplicated)”, “Fragmented”, or “Missing”, providing a standardized completeness score across all annotations (Figure S4G–I).

#### TFRecord generation of one-hot encoded genomic windows with exon and repeat masks

We loaded the repetitive, homologous, and paralogous-cleaned 16,384 bp windows with pysam (http://code.google.com/p/pysam/). For each window we: (1) one-hot encoded the DNA sequence; (2) computed exon masks by projecting GTF-derived transcript models onto the window and trimming a 2-bp flanking “chew” region; (3) derived repeat masks by flagging lowercase bases; and (4) assigned a species index. We flattened the resulting arrays (“sequence”, “exon_mask”, “repeat_mask”, “species”) to bytes and serialized them as TensorFlow Example protocol buffers. Finally, we wrote ZLIB-compressed TFRecord shards containing 32 examples each.

#### Shorkie LM training

We trained and evaluated Shorkie LM using the ZLIB-compressed TFRecord shards described above via a custom version of the baskerville API, called baskerville-yeast. We split shards 80:20 into train:validation. Each epoch sampled up to 150 minibatches (batch size = 8) from an in-memory shuffle buffer of 256 records, with full reshuffling between epochs.

At each step, we masked 15% of positions per sequence (m=⌊0.15×16,384⌋=2,457). Following the BERT protocol^110^: 80 % of masked sites were properly masked, 10 % were substituted by a random nucleotide, and 10 % were left unchanged to prevent the model from over-relying on the mask token distribution. To exploit the fact that double-stranded DNA is symmetric under reverse complementation, we applied reverse-complement augmentation to each input sequence with probability 0.5, thereby encouraging the model to learn strand-invariant features, a strategy shown to substantially improve performance^111,112^.

We computed categorical cross-entropy over masked positions only and reweighted the loss by genomic region. In order to focus the model on regulatory sequences, we down-weighted exonic and repeat regions by a factor of 0.1, following the success of this strategy for repeats in prior work^52^. We trained the model using the Adam optimizer^113^ ($learning\;rate\;=1\times 10^{-4};\;\beta_{1}=0.7;\;\beta_{2}=0.9$), global clipnorm = 0.1, and a linear warmup over the first 20,000 steps.

Training proceeded for up to 10,000 epochs (minimum = 100; maximum = 10,000), each capped at 150 steps, with early stopping according to the validation loss (patience = 1,000 epochs). At the end of each epoch, we averaged validation loss over five independent mask-and-predict passes per example (repeat_eval = 5) to reduce sampling noise. We retained the checkpoint with the lowest validation loss as the final model.

#### Shorkie LM evaluation

We evaluated held-out test windows using the same “mask → predict → tile” procedure. For each test sequence, we iteratively masked 15 % of positions (2,457 positions) and predicted them until all positions had been covered. We specified the --rc flag to average predictions from both the forward sequence and its reverse complement. We then computed the per-position cross-entropy loss (Equation 1) for each 16k window s as:

Equation 1 $${ℒ}_{CE_{s}}=\frac{\sum_{j=1}^{L}\;\left[-\sum_{n\in \{A,C,G,T\}}\;I_{j,n}^{\left(s\right)}ln\;p_{j,n}^{\left(s\right)}\right]w_{j}^{\left(s\right)}}{\sum_{j=1}^{L}\;w_{j}^{\left(s\right)}}$$

where L=16,384 is the window length, $I_{j,n}^{\left(s\right)}\in \{0,1\}$ is the one-hot indicator for base n at position j in window $s,\;p_{j,n}^{\left(s\right)}$ is the model’s predicted probability for base n, and $w_{j}^{\left(s\right)}$ is a position-specific weight (exon/repeat scaling) at that position. The global test loss is the average over all N test sequences:

Equation 2 $${ℒ}_{CE}=\frac{1}{N}\sum_{s=1}^{N}{ℒ}_{CE_{S}}$$

Perplexity is then defined as

Equation 3 $$\text{Perplexity}=exp\left({ℒ}_{CE}\right)$$

Finally, we calculated taxon-specific evaluation metrics by summing segment losses for each species and dividing by the number of segments for that species, yielding a species-level loss. In addition to loss and perplexity, we computed and stored a position probability matrix (PPM) for each sequence (x_pred), alongside one-hot inputs (x_true), species labels, and scaling weights for each base pair in a pred.npz file. Finally, we converted these PPMs into information content matrices (ICMs)^114,115^ to facilitate the identification of TF binding sites.

### Computation of Shorkie LM’s information content matrix

Let $p_{j,n}^{\left(s\right)}$ be the PPM probability for nucleotide n∈{A,C,G,T} at position j in the 16,384 bp window s. We transform this PPM into an ICM via the following steps. To avoid zeros, we first add a small pseudocount ε, yielding

Equation 4 $${\tilde{p}}_{j,n}^{\left(s\right)}=p_{j,n}^{\left(s\right)}+\varepsilon$$

and then renormalize across nucleotides so that

Equation 5 $${\overset{‾}{p}}_{j,n}^{\left(s\right)}=\frac{{\tilde{p}}_{j,n}^{\left(s\right)}}{\sum_{m\in \{A,C,G,T\}}\;{\tilde{p}}_{j,m}^{\left(s\right)}},\sum_{n}{\overset{‾}{p}}_{j,n}^{\left(s\right)}=1$$

The Shannon entropy at position j is

Equation 6 $$H_{j}^{\left(s\right)}=-\sum_{n\in \{A,C,G,T\}}{\overset{‾}{p}}_{j,n}^{\left(s\right)}{log}_{2}({\overset{‾}{p}}_{j,n}^{\left(s\right)})$$

with larger $H_{j}^{\left(s\right)}$ indicating greater nucleotide diversity (i.e. lower conservation). We then define the per-position information content as

Equation 7 $$C_{j}^{\left(s\right)}={log}_{2}(4)-H_{j}^{\left(s\right)}=2-H_{j}^{\left(s\right)},C_{j}^{\left(s\right)}\in [0,\;2]$$

For logo visualization, each nucleotide’s column height is set to

Equation 8 $$h_{j,n}^{\left(s\right)}={\overset{‾}{p}}_{j,n}^{\left(s\right)}\times C_{j}^{\left(s\right)},\;\sum_{n}h_{j,n}^{\left(s\right)}=C_{j}^{\left(s\right)}$$

Stacking letters in ascending $h_{j,n}^{\left(s\right)}$ order produces a DNA logo whose total column height reflects the information content, $C_{j}^{\left(s\right)}$, and whose individual letter heights encode the normalized nucleotide probabilities, ${\overset{‾}{p}}_{j,n}^{\left(s\right)}$.

### Constructing a DNA logo of the SMT3 promoter region using SpeciesLM

We adapted the Python notebook workflow of SpeciesLM^52^ (https://github.com/gagneurlab/dependencies_DNALM/blob/main/compute_and_visualize_dep_maps.ipynb). First, we loaded a pretrained BERT-style masked-language model (BertForMaskedLM; “johahi/specieslm-fungi-upstream-k1”) and its AutoTokenizer via Hugging Face Transformers^116^. Following the notebook, we applied this model to the 1 kb upstream region of SMT3 in *S. cerevisiae*. For each sequence, we tokenized the nucleotides and prepended a proxy-species identifier. At inference, a softmax produced per-position base-probability distributions. We then computed per-position information content (IC) from the reference (unmutated) probabilities against the yeast genomic background. Finally, we generated a sequence logo by setting the height of nucleotide b at position j to its predicted probability multiplied by the IC (Equation 8), so total stack height reflects conservation and relative letter heights encode base preferences (Figure 2B).

### Six genomic datasets and PPM construction for Shorkie LM evaluation

We evaluated Shorkie LM across six diverse genomic datasets:

1. *S. cerevisiae* reference genome (R64).
2. Four randomly selected *S. cerevisiae* strains: *S. cerevisiae* YJM1202, YJM1400, YJM555, and YJM984.
3. Five genomes within the Saccharomycetales order: *Candida albicans*, *Eremothecium gossypii* FDAG1, *Kluyveromyces lactis* str. NRRL Y-1140, *Komagataella phaffii* CBS 7435, and *Candida glabrata*.
4. Four genomes from the broader Ascomycota phylum: *Aspergillus fumigatus*, *Neurospora crassa*, *Penicillium chrysogenum* str. P2niaD18, and *Tuber melanosporum*.
5. Four genomes from the Orbiliales order: *Arthrobotrys flagrans* str. CBS H-5679, *Arthrobotrys oligospora* ATCC 24927, *Dactylellina haptotyla* CBS 200.50, and *Drechslerella stenobrocha* 248.
6. Four genomes from the Schizosaccharomycetales order: *Schizosaccharomyces cryophilus*, *Schizosaccharomyces japonicus*, *Schizosaccharomyces octosporus*, and *Schizosaccharomyces pombe*.

Except for *S. cerevisiae* and other Saccharomycetales genomes, all others were held out during training. We then applied the approach described in the “Shorkie LM Data Preprocessing” section to segment genomes into 16,384 bp windows, create ZLIB-compressed TFRecord shards, and predict with Shorkie LM.

### Motif discovery with TF-MoDISco-Lite

#### TF-Modisco run on the *S. cerevisiae* genome

To identify salient sequence motifs from Shorkie LM’s predictions, we employed TF-MoDISco-Lite (https://github.com/jmschrei/tfmodisco-lite), a memory- and time-efficient reimplementation of TF-MoDISco^53,54^. First, we loaded one-hot encoded inputs (x_true) and predicted probabilities (x_pred) from the pred.npz file generated during Shorkie LM evaluation. For each nucleotide channel i∈{A,C,G,T} at every position j=1,…,L within a 16,384 bp window, we added a pseudocount $\varepsilon =1\times 10^{-4}$ to each probability $p_{i,j}$. We then computed the local background frequency at position j as

Equation 9 $${\overset{‾}{p}}_{j}=\frac{1}{4}\sum_{i\in \{A,C,G,T\}}\left(p_{i,j}+\varepsilon \right)$$

And transformed each adjusted probability into a log-odds score:

Equation 10 $$\Delta_{i,j}=\left(p_{i,j}+\varepsilon \right)log\left(\frac{p_{i,j}\;+\;\varepsilon }{{\overset{‾}{p}}_{j}}\right)$$

thereby accentuating deviations from the local background. We saved these log-odds matrices as “x_true.npz” and “x_pred.npz”, reshaped them to (samples × positions × channels), and then ran modisco motifs s x_true.npz -a x_pred.npz -n 1000000 -w 16384 to sample one million seqlets across all 16,384 bp windows. TF-MoDISco-Lite then clustered those seqlets into consolidated motifs by first computing cosine similarity on gapped k-mer representations and next refining clusters via fine-grained realignment. For each resulting cluster, it computes both a contribution weight matrix (CWM) and a PWM. The modisco report step then generated motif logos and an interactive HTML summary.

#### Curating known TF-binding motifs from multiple yeast motif databases

We curated known TF-binding motifs from six publicly available databases, covering literature-curated associations, computational predictions, and high-throughput assay-derived specificity profiles. We included YEASTRACT, which provides 732 curated yeast motifs (average width 9.8 bp)^59^ (https://yeastract.com/); SwissRegulon Yeast, with 158 genome-wide motifs^58^ (https://swissregulon.unibas.ch/pages/); UniPROBE Yeast (GR09), containing 89 PBM-derived motifs^57^ (http://thebrain.bwh.harvard.edu/uniprobe/); MacIsaac v1, offering 124 phylogenetically conserved motifs^56^ (https://fraenkel-nsf.csbi.mit.edu/improved_map/); SCPD, supplying 24 promoter-derived motifs (https://esefinder.ahc.umn.edu/cgi-bin/tools/ESE3/esefinder.cgi we batch-normalize)^60^; and YeTFaSCo, a comprehensive compendium of 1,709 motifs for 256 yeast proteins calculated and quality-assessed by multiple metrics (http://yetfasco.ccbr.utoronto.ca/)^55^. All motifs were downloaded in MEME format from the respective websites and merged to obtain a comprehensive yeast TF-binding motif database for integration into our downstream analyses.

#### Genome-wide enrichment analysis of TF-MoDISco motifs around TSS

To map TF-MoDISco-derived motifs to the genome coordinates and assess their proximity to transcription start sites (TSS), we first parsed the HDF5 output from the TF-MoDISco-Lite run (modisco_results.h5) to extract seqlet positions (start, end, example index, strand) for each pattern. We then converted these coordinates to genomic coordinates by adding the seqlet offsets to the corresponding entries in the original BED file of one-hot input windows, yielding a unified BED of motif hits annotated with species, chromosome, start, end, strand, and the best-matching known motif with its associated q-value.

Next, we generated a comprehensive TSS annotation by parsing GTF files. For each gene, we recorded the the 5′ end of its transcripts (start coordinate for “+” strand, end for “−” strand) and sorted these TSS positions per chromosome. For each motif hit, we computed its midpoint and located the nearest TSS via binary search on the sorted list of transcripts, defining signed distance as negative for upstream (“−”) and positive for downstream (“+”) relative to the TSS strand.

To establish a background distribution, we sampled one random position per seqlet from the metaclusters on the same chromosome, drawing positions uniformly across chromosome lengths, and calculated each position’s closest-TSS distance using the same procedure. Finally, we compared the observed and background distance distributions by plotting histograms over a ±2.5 kb window centered on the TSS to highlight motif enrichment patterns.

#### Embedding t-SNE clustering using Shorkie LM

To generate low-dimensional embeddings of genomic contexts, we first defined three interval classes across all sixteen *S. cerevisiae* chromosomes: (1) Promoters: 500 bp immediately upstream of each start codon; (2) gene bodies: the span between each gene’s start and end coordinates; (3) intergenic regions: all regions not annotated as gene, exon, or CDS.

Each interval was retrieved from the reference genome using pysam, centered, and end-padded to 16,384 bp. Sequences on the “−” strand were reverse-complemented. We then one-hot encoded the four nucleotide channels and concatenated a 165-dimensional species one-hot vector, setting the *S. cerevisiae* channel (index 114) to 1. Batches of eight intervals (shape 16,384×170) were fed into a pretrained Shorkie LM. To capture intermediate representations, we defined for each selected layer a sub-model that takes the original LM inputs and returns that layer’s activations. Ten selected layers are max_pooling1d_6, multihead_attention, dense, dense_1, multihead_attention_7, dense_14, dense_15, dense_16, dense_28, dense_29.

For each interval, we mean-pooled the per-position outputs across the sequence axis, concatenated the resulting vectors to form an embedding of dimension D, and stored the matrix (N intervals × D) in HDF5 datasets. We also saved accompanying metadata arrays: chromosome, coordinates, strand, feature class, and gene_id. Next, we aggregated metadata across all chromosomes and parsed each gene interval’s biotype from the Ensembl GTF, categorizing intervals into five groups: “Protein-coding gene,” “Intergenic region,” “tRNA,” “Transposable element,” and “Promoter”. For each layer’s embedding matrix, we applied t-SNE^117^ (scikit-learn TSNE with n_components = 2) to project the D-dimensional embeddings into two dimensions. The resulting 2D coordinates were visualized to assess clustering patterns by genomic feature.

### Shorkie and Shorkie_Random_Init data pre-processing

#### RNA-Seq perturbation experiments

The design of the inducible genetic perturbation experiments builds on methods developed for the Induction Dynamics gene Expression Atlas^34^, where hundreds of transcription factors were independently induced and resultant gene expression changes were profiled over time using RNA hybridization microarrays. New data used for Shorkie-supervised training were generated at Calico Life Sciences LLC using updated miniaturized chemostats, or ministats. The instrumentation and RNA-sequencing protocols are described here.

#### Strain construction and selection

Each time-course experiment uses a strain selected from the Yeast Estradiol strains with Titratable Induction (YETI) collection^118^, where the native promoter for a gene of interest was replaced with a synthetic Z3EV inducible promoter which drives transcription in the presence of estradiol. New data collected for this study can be split into three partitions: (1) many replicates of pre-induction cultures of the MSN4 inducible strain, (2) a set of 8 TF perturbations in replicate, matched to previously measured microarray data, and (3) a set of 460 other genes, including kinases, phosphatases, and other transcriptional regulators prioritized as likely to have downstream transcriptional changes as assessed using evidence from YeastMine curated annotations^119^, Phenome^120^, and Fitness Clusters ^121^.

#### Growth conditions

For all experiments, cells were grown under continuous culturing conditions. Cultures were maintained under phosphate limitation in minimal chemically defined media prepared by mixing 20mL of 1000x vitamin solution, 20mL of 1000x metals, 40mL of 10g/L KH2PO4, 1L 40% dextrose, and 2L 10X salts solution in 16L of milliQ water to bring the total volume to 20L. (Stock solutions defined in Table S1).

#### Instrumentation and perturbation experiments

To increase throughput and coverage of gene expression changes, we developed a ministat array system capable of growing 24 30mL cultures in parallel to steady state, applying a chemical perturbation, and generating samples for time-resolved omic measurements. The system is housed in a 30°C warm room and consists of four banks of six 100 mL round-bottom vessels, integrated via a 24-vessel manifold into a complete ministat array (Figure S11).

Each ministat array incorporates three commercial peristaltic pumps: (1) a media pump that delivers fresh media to each vessel at a constant rate (the dilution rate), (2) a sampling pump that extracts a bolus of cells from each culture, and (3) an input pump used initially to inoculate cultures and subsequently to deliver a chemical perturbation (e.g., estradiol). Each pump uses separate tubing for each of the 24 ministats. Each vessel is also equipped with a tube for the effluent, which is continuously weighed to monitor and control the dilution rate, and a tube for delivering air. Air is supplied via a Flow Master 2400 airflow regulator connected to a humidifier to minimize culture evaporation. The regulator also splits the airflow into 24 individually adjustable lines, one for each culture.

For each culture, a pre-induction ($T_{0}$) sample was collected prior to perturbation. The input pump then delivered 100 μL of 500 μM b-estradiol in base media from a 96-well plate into each vessel to initiate the time course. Following induction, cultures were typically sampled at 8 timepoints using the sampling pump to collect each sample into 96 deep-well plates pre-filled with chilled lysis buffer containing RNase inhibitor (Takara). Samples from the 24 ministats were staggered across quadrants of each 96-well plate, enabling systematic re-arraying of the eight timepoint-specific plates into two consolidated 96-well plates for downstream processing. Sample plates were flash frozen in liquid nitrogen immediately after collection.

#### RNA-seq library preparation

Samples from four timepoints were combined into a single 96-well PCR plate using the Bravo BenchCel system and processed using a miniaturized, high-throughput adaptation of standard library preparation protocols. After addition of oligo-dT primer (sequence: AAGCAGTGGTATCAACGCAGAGTACTTTTTTTTTTTTTTTTTTTTTTTTTTTTTTVN), samples were lysed by three freeze-thaw cycles and incubated at 42°C for 3 minutes. cDNA synthesis was performed using the SMARTScribe Reverse Transcriptase kit (Takara), with 2.4 μM LNA template switch oligo (sequence: AAGCAGTGGTATCAACGCAGAGTACrGrG+G) and RNase inhibitor. For cDNA amplification, SeqAmp DNA polymerase (Takara) and ISO PCR primer (sequence: AAGCAGTGGTATCAACGCAGAGT) were added. The samples were placed in a thermocycler with the following conditions: 95°C for 1 min, 10 cycles of [98°C for 10 s, 65°C for 30 s, 68°C for 3 min], 72°C for 10 min. Cleanup was performed using RNAclean XP beads (Beckman Coulter) at 0.9x reaction volume.

Relative cDNA concentration was measured using Quant-iT PicoGreen (Fisher). For absolute quantification and quality control, 11 representative samples (chosen to span the range of PicoGreen values) were assessed using a High Sensitivity DNA kit (Agilent) on the BioAnalyzer. These values were used to generate a standard curve, and all samples were diluted to 200 pg/μL using a Mantis dispenser.

Library preparation was performed using the Nextera XT kit on cDNA from two ministat batches at a time, re-arrayed into a 384-well PCR plate. Samples were pooled, and pooled libraries underwent a double RNAclean XP bead cleanup. Final library concentration and quality were confirmed using a High Sensitivity DNA kit. Libraries were sequenced by Genewiz on the Illumina NovaSeq.

#### *S. cerevisiae* R64 reference genome pre-processing

Contigs from *S. cerevisiae* R64 reference were first split at assembly gaps and at hypervariable regions identified in the Rossi et. al. study (these sites include the rDNA locus, tRNA genes and telomere regions and are available in 02_References_and_Features_Files at https://github.com/CEGRcode/2021-Rossi_Nature) hereafter referred to as rossi_mask.bed.

We then trimmed 1,024 bp from each contig end and discarded those shorter than 16,384 bp. Contigs longer than 786,432 bp were split in half. The genome was then segmented into overlapping 16,384 bp windows with a 6,165 bp stride. These windows were shuffled and partitioned into eight cross-validation folds by balancing total nucleotide counts. Unmappable positions (from hypervariable regions described in rossi_mask.bed) were annotated, and any window with > 50% unmappability (--umap_clip 0.5) was removed.

#### ChIP-exo and ChIP-MNase samples preprocessing

Paired-end sequencing data was obtained directly from Rossi et al^65^ (GSE147927) and sequence alignment was performed using Bwa-0.7.17 mem algorithm^122^ and multi-mappers removed using SAMtools^123^. For ChIP-exo data the position of the 5’ end of Read 1 was used, while the full span for MNase was used to generate tracks. BAM files were additionally qc’d using PICARD (https://github.com/broadinstitute/picard) to mark and remove duplicates. Reads overlapping regions described in rossi_mask.bed were removed and experiments with less than 10,000 remaining reads and greater than 75 percent duplication rate were dropped from the dataset. Lastly, BEDtools^124^ and bedGraphToBigWig (https://www.encodeproject.org/software/bedgraphtobigwig/) were used to transform the BAM files to BigWig tracks, yielding 1,128 ChIP-exo, and 20 ChIP-MNase tracks.

#### RNA-seq samples preprocessing

First adapter sequences were trimmed from the FASTQ files using bbmap (https://github.com/BioInfoTools/BBMap). Then transcript alignment and quantification was performed using STAR^125^. A genomic index was created using S288c_R63–3 and quantification was performed using GeneCounts with the following parameters (--outFilterMultimapNmax 1, --bamRemoveDuplicatesType UniqueIdentical, --alignIntronMin 10, --alignIntronMax 2500, --alignMatesGapMax 2500). BAM files were processed to remove PCR duplicates marked using PICARD^126^. Lastly, bam_cov.py (https://github.com/calico/basenji/blob/master/bin/bam_cov.py) was used to produce BigWig tracks. Data from 1000 strains (Caudal et al.) used unpaired RNA-seq^66^, and was filtered to retain samples with greater than 150,000 reads and less than 80 percent duplication rate while the in-house generated induction experiments used paired-end RNA-seq and was filtered to retain samples with greater than 150,000 reads and mean insert size greater than 250 bp. This produced 3,053 induction RNA-Seq tracks and 1,014 1,000-strain RNA-Seq tracks.

#### Validation

To ensure that gene expression dynamics measured using the new ministat array system are reliable and accurate, we compared data collected on the high-throughput ministat array to previously collected microarray data from Hackett et al.^34^. To assess the correspondence between transcriptional profiles in each system, we calculated the Pearson correlation of gene expression fold-changes across all genes for a given matched timepoint post induction (Figure S12A), using both raw and log2 shrunken fold-changes. Next, to evaluate the new system’s sensitivity in detecting previously identified differentially expressed genes, we conducted an ROC analysis. To select a positive set of differentially expressed genes, we selected genes with an absolute log_2_ fold-change greater than one in the microarray data as a heuristic (Figure S12B). We then tested for differential expression in the new system by separately fitting a standard ordinary least squares linear regression for each gene’s expression time course following perturbation. In this model, gene expression relative to the mean $T_{0}$ expres-sion is described as a function of the time point, coded as a categorical variable for each timepoint, and a co-variate for the culture vessel corresponding to experiment replicate. Then we fit an ANOVA for each gene expression time course regression and used the F-statistic, describing the variance between timepoints to the variance among replicates within a timepoint. By varying the cutoff F-statistic, we calculated the AUROC (Figure S12C-D).

#### Track data transformation

We processed 5,215 BigWig tracks: 3,053 induction timepoint RNA-Seq datasets^34^, 1,014 RNA-Seq datasets from various yeast strains^66^, 1,128 ChIP-exo tracks, and 20 ChIP-MNase tracks^65^. For each 16,384-bp window, we extracted per-base coverage and imputed missing values with the window’s median. To reduce edge effects, we cropped 1,024 bp from each end, retaining a 14,336-bp interior. We then summed coverage across consecutive 16-bp intervals to form 896 non-overlapping bins, yielding a 896-element vector per window. We saved these vectors as float16 in HDF5. Finally, we serialized the one-hot DNA sequence (via pysam), the corresponding binned coverage vector, and the unmappability mask into ZLIB-compressed TFRecord files (256 windows per file) organized by cross-validation fold.

#### Shorkie model architecture and hyperparameters

Shorkie fine-tunes the pretrained Shorkie LM backbone (13.7 M parameters) to predict RNA-Seq, ChIP-exo, and ChIP-MNase signals across 16,384 bp windows. Its trunk replicates Shorkie LM exactly:

- Initial Conv1D projection: 11 bp kernel × 96 filters (linear activation)
- Seven residual down-sampling blocks: each block is BatchNorm→GELU→Conv1D(5 bp) with filter counts increasing from 96 to 384 in 32-filter steps, followed by 5% dropout, a skip connection, and MaxPool1D (pool size = 2)
- Transformer bottleneck: eight layers operating over 128 positions. Each layer contains LayerNorm→4-head self-attention (model dimension = 384, key dimension = 64) with 20% dropout, followed by a feedforward (LayerNorm→Dense→ReLU→Dropout→Dense→Dropout) and residual connections.

The decoder uses the same U-Net up-sampling scheme as Shorkie LM but with only three up-sampling stages to restore the 16 bp resolution. At each stage, feature maps are batch-normalized, passed through a GELU nonlinearity, projected via a Dense layer (384 → 384), and doubled in length with UpSampling1D, then merged with the corresponding encoder output via a U-Net skip connection. At the final 16 bp resolution, a Cropping1D layer (cropping = 64) removes convolutional padding artifacts and is followed by GELU. A single Dense layer then projects each position into 5,215 channels: TF-perturbed RNA-Seq (n = 3,053), 1,000-strain RNA-Seq (n = 1,014), ChIP-exo (n = 1,128), and ChIP-MNase histone marks (n = 20), and a Softplus activation ensures all outputs remain positive.

We fine-tuned Shorkie with Adam ($\beta_{1}=0.7,\;\beta_{2}=0.9$; global clip-norm = 0.1), $learning\;rate=2\times 10^{-5}$ with 20,000 warm-up steps, batch = 8, using a Poisson + Multinomial loss (5x scaling of multinomial loss component) and early stopping with a patience of 150 epochs.

### Shorkie_Random_Init model architecture and hyperparameters

The Shorkie_Random_Init model uses an identical architecture as Shorkie but with all weights initialized from scratch. We trained Shorkie_Random_Init in a supervised manner using Adam ($\beta_{1}=0.9,\;\beta_{2}=0.999$; global clip-norm = 0.1), learning rate = 1 × 10^−4^ with 5,000 warm-up steps, and otherwise identical hyperparameters. Shorkie_Random_Init thus serves as a baseline for quantifying the performance improvements attributable to transfer learning.

### Shorkie and Shorkie_Random_Init bin-level, gene-level and track-level evaluation Bin-level evaluation

We generated per-base predictions for each 16,384 bp input window by first averaging the model’s outputs across both the forward and reverse-complement strands (strand-ensemble) and then applying a shift-ensemble over offsets S={0,1} bp to smooth boundary artifacts. Specifically, the ensemble prediction at genomic position i and track j is defined as:

Equation 11 $${\overset{ˆ}{y}}_{i,j}^{\left(ens\right)}=\frac{1}{\left|\;S\right|}\sum_{s\in \{0,1\}}\frac{y_{i\;+\;s,j}^{fwd}+y_{i+s,j}^{rc}}{2}$$

where $y_{i,j}^{fwd}$ and $y_{i,j}^{rc}$ are the strand-specific model outputs. We compared ${\overset{ˆ}{y}}_{i,j}^{\left(ens\right)}$ to measured coverage $y_{i,j}$ and reported bin-level Pearson correlation and coefficient of determination ($R^{2}$) across all tracks and cross-validation folds.

### Raw (non-normalized) gene-level evaluation

Let G denote all annotated genes from the *S. cerevisiae* GTF. We assigned a given 16,384 bp bin i to gene g if at least 50% of the bin’s width (p) overlaps and exon of gene g:ℬ(g)={b:overlap(b,g)≥0.5p}.

For each gene g and track j, we aggregated the strand- and shift-ensemble predictions ${\overset{ˆ}{y}}_{i,j}^{\text{(ens)}}$ and the true coverages $y_{i,j}$ over ℬ(g) and stabilized variance via a ${log}_{2}$-transform with a pseudocount of 1:

Equation 12 $${\hat{Y}}_{g,j}={log}_{2}\left(\sum_{i\in ℬ(g)}\;{\overset{ˆ}{y}}_{i,j}^{\left(ens\right)}+1\right),Y_{g,j}={log}_{2}\left(\sum_{i\in ℬ(g)}\;y_{i,j}+1\right)$$

For each track j, we computed gene-level Pearson r and $R^{2}$ between ${\left\{Y_{g,j}\right\}}_{g\in G}$ and ${\left\{{\overset{ˆ}{Y}}_{g,j}\right\}}_{g\in G}$.

### Assay-normalized gene-level evaluation

To account for the differing dynamic ranges across assays, we first partition tracks into assay-specific groups $T_{k}$, including TF-perturbed RNA-seq, 1,000-strain RNA-seq, ChIP-exo, and ChIP-MNase. Within each group $T_{k}$, we independently applied quantile normalization (QN) to the ${log}_{2}$-transformed aggregated coverages to equalize their distributions:

Equation 13 $${\overset{ˆ}{Z}}_{g,j}={QN}_{k}({\hat{Y}}_{g,j}),\;Z_{g,j}={QN}_{k}\left(Y_{g,j}\right),\;\forall j\in T_{k}$$

We then mean-centered each gene within its assay group:

Equation 14 $$\Delta {\overset{ˆ}{Z}}_{g,j}={\overset{ˆ}{Z}}_{g,j}-\frac{1}{\left|T_{k}\right|}\sum_{j^{\prime }\in T_{k}}{\overset{ˆ}{Z}}_{g,j^{\prime }},\;\Delta Z_{g,j}=Z_{g,j}-\frac{1}{\left|T_{k}\right|}\sum_{j^{\prime }\in T_{k}}Z_{g,j^{\prime }}$$

We summarized the gene-level concordance with Pearson r and $R^{2}$ between ${\left\{\Delta Z_{g,j}\right\}}_{g\in G}$ and ${\left\{\Delta {\overset{ˆ}{Z}}_{g,j}\right\}}_{g\in G}$ for each j. Normalizing by assay-specific variability yields metrics that isolate predictive skill from absolute signal scale and enable evaluation of track specificity—that is, whether the model reproduces the differences among tracks within each assay.

### Within-gene bin-level consistency

To evaluate how precisely Shorkie reconstructs fine-scale positional coverage within individual genes, we analyzed consistency at the bin-level within each gene. First, for each gene g and track j, we derived ${log}_{2}$-transformed coverage vectors from the predictions and measured coverages:

Equation 15 $${\overset{ˆ}{v}}_{g,j}^{raw}={\left\{{log}_{2}\left({\overset{ˆ}{y}}_{i,j}^{\left(ens\right)}+1\right)\right\}}_{i\in B(g)},\;v_{g,j}^{raw}={\left\{{log}_{2}\left(y_{i,j}+1\right)\right\}}_{i\in B(g)}$$

We computed Pearson r for gene-track pairs with non-trivial variation (both variances $>10^{-6}$). To de-emphasize nearly flat profiles, we further required $Var\left({\overset{ˆ}{v}}_{g,j}^{\text{raw}}\right)>\tau_{j}$, where $\tau_{j}$ is the 80 th percentile of predicted variances across genes for track j. For each gene we summarized:

$$r_{g}^{\text{within,}\;\text{raw}\;}=\frac{1}{\left|\left\{j:Var\left({\overset{ˆ}{v}}_{g,j}^{\text{raw}\;}\right)\;>\;\tau_{j}\right\}\right|}\sum_{j:Var\left({\overset{ˆ}{v}}_{g,j}^{\text{raw}\;}\right)>\tau_{j}}r_{g,j}^{\text{bin,}\;\text{raw}\;}$$

An identical procedure was conducted using quantile-normalized and mean-centered vectors to compute $r_{g}^{\text{within,}\;\text{norm}}$.

### Track-level evaluation

To evaluate Shorkie’s ability to accurately capture each gene’s pattern across RNA-Seq assays specifically, we performed a track-level analysis focusing exclusively on RNA-Seq tracks. For each gene g, we constructed vectors of coverage across all RNA-Seq tracks, both in their raw and normalized (mean-centered and quantile-normalized) forms:

Equation 16 $$Y_{g}={\left\{Y_{g,j}\right\}}_{j\in J_{RNA-Seq}},\;{\overset{ˆ}{Y}}_{g}={\left\{{\overset{ˆ}{Y}}_{g,j}\right\}}_{j\in J_{RNA-Seq}},\;\Delta Z_{g}={\left\{\Delta Z_{g,j}\right\}}_{j\in J_{RNA-Seq}},\;\Delta {\overset{ˆ}{Z}}_{g}={\left\{\Delta {\overset{ˆ}{Z}}_{g,j}\right\}}_{j\in J_{RNA-Seq}}$$

We reported Pearson r and $R^{2}$ between predicted and observed vectors for both raw ($Y_{g}$ and ${\overset{ˆ}{Y}}_{g}$) and normalized ($\Delta Z_{g}$ and $\Delta {\overset{ˆ}{Z}}_{g}$) settings, summarizing per gene.

## Attention weight matrix visualization from selected Shorkie embeddings

To inspect how Shorkie’s transformer blocks focus on different parts of a gene, we extracted 16,384 bp windows centered on two example genes (EFM5 at chr VII: 489,391–505,775; RPL7A at chr VII: 356,973–373,357) from the *S. cerevisiae* R64 reference using pysam. We center-trimmed or padded each window with “N”s to exactly 16,384 bp and parsed the Ensembl GTF with PyRanges^127^ to build an annotation table of gene bodies, exons, and 5’/3’ UTRs for downstream overlay.

We evaluated three models: Shorkie LM (pretrained LM), Shorkie (fine-tuned), and Shorkie_Random_Init (baseline). For each 16,384 × 4 input tensor, we averaged forward and reverse-complement predictions and concatenated results across replicates to yield a 1 × N_reps × 16,384 coverage array. To capture self-attention, we computed dot-products between learned query and key vectors at every position pair (i,j), applied softmax to obtain attention weights, and collected these across all eight cross-validation folds for (i) the first transformer block and (ii) the final two blocks. This yielded a single 128×128 attention map per block set.

For visualization, we clipped each fold-averaged attention map below 10^−4^ and above 0.05 to enhance contrast, then displayed it as a heatmap across the full window. We plotted predicted coverage as filled curves along the top and right margins. We converted genomic features into “attention-bin” coordinates (bin index = ⌊(position − window_start)/128⌋) and overlaid as colored boxes for gene bodies or lines for UTRs and exons, with strand-specific coloring.

## *In silico* mutagenesis analysis of Shorkie and Shorkie_Random_Init

### Sequence extraction and formatting

We applied *in silico* mutagenesis (ISM) to quantify the effect of every single-nucleotide variant (SNV) within yeast promoter regions based on Shorkie and Shorkie_Random_Init predictions. We defined input regions as 16,384-bp windows centered on promoter segments (450 bp upstream and 50 bp downstream of the TSS). The promoter set consisted of 137 ribosomal protein genes, 64 ribosome/rRNA biosynthesis (RRB) genes, and 3,258 additional protein-coding genes from the *S. cerevisiae* R64 reference genome. We extracted the corresponding sequences from the reference FASTA while preserving strand orientation.

For each 16 kb window, we computed ISM maps across the 500 bp promoter segment. For each reference sequence s and position p=1,…,500, we generated three mutant sequences by substituting the reference base with each of the three alternative nucleotides n∈{A,C,G,T}\{ref}, yielding 3 × 500 mutant sequences per window.

### ISM importance score matrix construction

We one-hot encoded all reference and mutant sequences and ran them through Shorkie and Shorkie_Random_Init with strand-ensemble averaging. For each sequence s, genomic bin i∈{1,…,896}, and track j, let ${\overset{ˆ}{y}}_{\text{ref},\;i,j}^{\left(s\right)}$ be the model prediction for the reference sequence, and ${\overset{ˆ}{y}}_{\text{alt},\;i,j}^{\left(s,p,n\right)}$ be the prediction for the variant at position p with nucleotide n. We computed the ${log}_{2}$ fold-change score to quantify the variant effect:

Equation 17 $${log}_{2}\;{FC}_{j}^{\left(s,p,n\right)}={log}_{2}\left(\sum_{i\in ℬ}\;{\overset{ˆ}{y}}_{alt,i,j}^{\left(s,p,n\right)}+1\right)-{log}_{2}\left(\sum_{i\in ℬ}\;{\overset{ˆ}{y}}_{ref,\;i,j}^{\left(s\right)}+1\right)$$

where ℬ indexes the 896 output bins. We saved the resulting log_2_ fold-change scores, together with metadata (chromosome, start, end, strand, reference, and alternate alleles), to an HDF5 file (scores.h5) via h5py (https://www.h5py.org/).

### ISM importance score matrix normalization and ISM map visualization

To derive per-position importance profiles, we defined the set J of $T_{0}$ RNA-Seq tracks. For each sequence s, mutation (p,n), and position i, we averaged the ${log}_{2}$ fold-change scores across $T_{0}$ tracks:

Equation 18 $$M_{i,n}^{\left(s\right)}=\frac{1}{\left|J\right|}\sum_{j\in J}{log}_{2}\;{FC}_{i,n,j}^{\left(s\right)}$$

where i indexes positions and n denotes nucleotides. Next, we zero-mean normalized at each position i across the four nucleotides:

Equation 19 $${\tilde{M}}_{i,n}^{\left(s\right)}=M_{i,n}^{\left(s\right)}-\frac{1}{4}\sum_{m\in \{A,C,G,T\}}M_{i,m}^{\left(s\right)}$$

Finally, to focus on reference-base contributions, we computed:

Equation 20 $${Logo}_{i}^{\left(s\right)}=\sum_{n\in \{A,C,G,T\}}{\tilde{M}}_{i,n}^{\left(s\right)}\times H_{i,n}^{\left(s\right)}$$

where $H_{i,n}^{\left(s\right)}\in \{0,\;1\}$ is the one-hot indicator for the reference base at position i. Finally, we visualized per-position scores ${Logo}_{i}^{\left(s\right)}$ as DNA sequence logos (ISM maps), highlighting the magnitude and direction of variant effects across the promoter window.

### Motif discovery using TF-MoDISco-Lite

To identify TF-binding motifs, we first constructed two arrays: ref.npz of shape ($N_{\text{seq}},4,M$), containing one-hot encoded reference sequences, and pred.npz of shape $\left(N_{\text{seq}},4,M,|J|\right)$, containing per-replicate variant scores for $T_{0}$ RNA-Seq tracks.

Here, $N_{\text{seq}}$ is the number of promoter windows, M=500 is the promoter window length, and |J| is the number of $T_{0}$ RNA-Seq replicates. We reshaped pred.npz to $\left(N_{\text{seq}}\times |J|,4,M\right)$ by concatenating across replicates, then ran TF-MoDISco-Lite (see “TF-MoDISco run on the S. cerevisiae genome” section) on these matrices to uncover recurring sequence motifs.

### Time-series motif analysis of TF-induction with Shorkie

To capture dynamic motif changes during TF induction (e.g., MSN2, MSN4, MET4), we filtered the RNA-Seq metadata to select only the tracks corresponding to each TF time course and grouped biological replicates by sampling time. We parsed time-point annotations (e.g., “T_0”, “T_15”, “T_30”) directly from the track identifiers, ordered them chronologically, and for each time point $T_{t}$ defined $J_{t}$ as the set of associated RNA-Seq track indices.

For each promoter window s, base position i∈{1,2,…,500}, nucleotide channel n∈{A,C,G,T}, and time point $T_{t}$, we computed an ISM importance score matrix by averaging log_2_ fold-change scores across all replicate tracks:

Equation 21 $$M_{i,n}^{\left(s,T_{t}\right)}=\frac{1}{\left|J_{t}\right|}\sum_{j\in J_{t}}{log}_{2}{FC}_{i,n,j}^{\left(s,T_{t}\right)}$$

To focus on differential saliency relative to baseline expression, we baseline-corrected each time point by subtracting the $T_{0}$ map:

Equation 22 $$\Delta M_{i,n}^{\left(s,T_{t}\right)}=M_{i,n}^{\left(s,T_{t}\right)}-M_{i,n}^{\left(s,T_{0}\right)}$$

We then applied zero-mean normalization across nucleotides at each position to ΔM and visualized the resulting ΔISM maps for each $T_{t}$. Finally, we reshaped per-timepoint ΔISM matrices to $\left(N_{\text{seq}}\times \left|J_{t}\right|,4,M\right)$ and applied TF-MoDISco-Lite to identify motifs whose importance trajectories changed over the induction time course.

### Gene-level coverage calculation for experimental measurements and Shorkie predictions

To compare experimental and predicted gene-level coverages at Shorkie’s native 16 bp resolution, we summed bin values over each gene’s span. For gene g and track j, let ℬ(g) be the set of overlapping 16 bp bins. We define:

Equation 23 $${cov}_{j}^{exp}(g)=\sum_{i\in ℬ(g)}y_{i,j}^{exp},\;{cov}_{j}^{pred}(g)=\sum_{i\in ℬ(g)}y_{i,j}^{pred}$$

where ℬ(g) indexes the bins overlapping gene g. Next, we normalized to reads-per-million (RPM) using each sample’s library size ${libsize}_{j}$,

Equation 24 $${cov}_{j}^{exp,\;RPM}(g)=\frac{{cov}_{j}^{exp}(g)}{{libsize}_{j}}\times 10^{6},\;{cov}_{j}^{pred,\;RPM}(g)=\frac{{cov}_{j}^{pred}(g)}{{libsize}_{j}}\times 10^{6}$$

To characterize time-dependent gene expression trajectories in TF-induction experiments, we averaged RPM-normalized coverages across replicate tracks at each time point $T_{t}$:

Equation 25 $${\bar{cov}}^{\left(T_{t},exp,RPM\right)}(g)=\frac{1}{\left|J_{t}\right|}\sum_{j\in J_{t}}{cov}_{j}^{exp,\;RPM}(g),\;{\bar{cov}}^{\left(T_{t},pred,RPM\right)}(g)=\frac{1}{\left|J_{t}\right|}\sum_{j\in J_{t}}{cov}_{j}^{\text{pred,}\;\text{RPM}}(g)$$

This yields matched, per-gene coverage trajectories for both experimental measurements and Shorkie predictions.

### Euclidean distance calculation of ISM maps

To quantify motif importance changes during induction, we extracted the normalized importance score matrix $P_{s,t}\in R^{L\times 4}$ for each promoter window s and timepoint t. We flattened this matrix into a vector $v_{s,t}\in R^{4L}$, and computed pairwise Euclidean distances between timepoints:

Equation 26 $$D_{s}\left(t_{1},t_{2}\right)={\left\|v_{s,t_{1}}-v_{s,t_{2}}\right\|}_{2},\;\forall \;t_{1},t_{2}\in \{1,\ldots ,T\}$$

To summarize across all promoter windows, we conducted element-wise averaging:

Equation 27 $$\overset{‾}{D}\left(t_{1},t_{2}\right)=\frac{1}{S}\sum_{S=1}^{S}D_{S}\left(t_{1},t_{2}\right)$$

where S is the total number of promoter windows. We visualized the resulting mean Euclidean-distance matrix $\overset{‾}{D}$ as a heatmap, effectively capturing motif shifts over the TF induction time course.

### *cis*-eQTL analysis with Shorkie and DREAM challenge models

To evaluate model performance on *cis*-eQTLs, we benchmarked Shorkie against the DREAM challenge models^93^ using two independent eQTL datasets.

### Caudal et al. eQTLs from the pan-transcriptome of ~1,000 yeast natural isolates

We imported the GWAS summary statistics^66^ (file: GWAS_combined_lgcCorr_ldPruned_noBonferroni_20221207.tab; downloaded from The 1002 Yeast Genome website: http://1002genomes.u-strasbg.fr/files/RNAseq) into a DataFrame and removed variants labeled as masked (ld_mask = “masked”) were. We normalized Phenotype (Pheno_pos) and SNP (ChrPos) positions by chromosome length, then classified a variant as *cis* if |ChrPos − Pheno_pos| ≤ 8,000 bp on the same chromosome and as *trans* otherwise. We further stratified variants by subtype (SNP vs. CNV) (Figure S20A).

We parsed the gVCF containing 1,011 yeast isolates (1011Matrix.gvcf; downloaded from http://1002genomes.u-strasbg.fr/files/)^95^ with pysam to extract reference and alternate alleles, chromosome, position, and quality. We then merged the *cis* and *trans* eQTL tables with the gVCF DataFrame on chromosome and position to identify intersecting and unique variant sets.

We removed variants with missing or non-positive P-values and computed significance as $-{log}_{10}$(PValue). Finally, we generated a Manhattan plot by plotting $-{log}_{10}$ (PValue) against cumulative genomic position with alternating colors per chromosome, overlaid a genome-wide significance line at $P=5\times 10^{-8}$, and placed chromosome ticks at median cumulative positions (Figure S20B).

### Kita et al. eQTLs from 85 diverse *S. cerevisiae* isolates

We imported the summary statistics (pnas.1717421114.sd01; downloaded from https://www.pnas.org/doi/suppl/10.1073/pnas.1717421114/suppl_file/pnas.1717421114.sd01.txt), yielding 1,640 eQTLs. From these, we selected 683 variants in four genomic contexts: Promoter, UTR5, UTR3, and ORF. For each variant, we computed the absolute distance between its genomic coordinate (ChrPos) and the target gene’s TSS, labeling it *cis* if |ChrPos − TSS| ≤ 8,000 bp and *trans* otherwise. Finally, we retrieved the corresponding allele sequences from 1011Matrix.gvcf using pysam.

### Negative-eQTL sampling

We generated four independent negative-eQTL sets by sampling common, non-coding variants that matched the positive eQTLs in allele composition and distance to the gene TSS. We extracted *S. cerevisiae* TSS coordinates from the Ensembl GTF. From the gVCF, we retained variants located outside CDS/exon intervals with allele frequency (AF) ≥ 0.05 and excluded any variant that matched a positive eQTL.

For each positive eQTL, we shuffled its matching candidate list of negative variants on the same chromosome and attempted to select one negative variant whose distance to a randomly chosen gene’s TSS matched the positive’s distance within ±100 bp (with a fallback window of ±200 bp if no match emerged). We enforced that each negative was used only once per iteration, yielding one negative per positive. We repeated this sampling four times to produce four distinct negative-eQTL sets.

### Variant effect prediction with Shorkie

We predicted how individual variants alter gene-level coverage profiles with Shorkie. For each variant, we (1) extracted a 16,384 bp window centered on the SNP; (2) verified that the reference allele matched the extracted sequence; (3) generated one-hot encodings for both the reference and alternate alleles; and (4) averaged predictions over forward and reverse-complement strands. Within the window, we summed the predicted coverage values over all bins overlapping the annotated gene g’s exons:

Equation 28 $${Cov}_{ref}=\sum_{i\in ℬ(g)}y_{ref}(i),\;{Cov}_{alt}=\sum_{i\in ℬ(g)}y_{alt}(i)$$

where ℬ(g) indexes the bins overlapping gene g. We then defined the log_2_ fold-change score as

Equation 29 $${log}_{2}\;{FC}_{\text{Shorkie}}={log}_{2}\left({Cov}_{alt}+1\right)-{log}_{2}\left({Cov}_{ref}+1\right)$$

### eQTL ISM analysis with Shorkie

To probe local sequence drivers, we applied the ISM pipeline to 80-bp windows centered on each SNP (to match DREAM input length), generated an 80×3Δ matrix per variant, formed the reference-average matrix, and rendered sequence logos.

### Predicting eQTL SNPs with DREAM challenge models

To benchmark Shorkie against established DREAM challenge models, we evaluated three pretrained models: convolutional (DREAM-CNN), recurrent (DREAM-RNN), and attention-based (DREAM-Atten), on both positive eQTLs and the four independently sampled negative sets.

### Sequence extraction and formatting

For each variant (positive or negative), we extracted an 80bp window centered on the SNP from the *S. cerevisiae* genome (40 bp upstream and 39 bp downstream), then prepended a fixed 17bp upstream flank (TGCATTTTTTTCACATC) and appended a 13 bp downstream flank (GGTTACGGCTGTT), following the DREAM models’ expected input format, yielding final input sequences of 110 bp.

### Model inference and log fold-change score calculation

We one-hot encoded the 110-bp reference and alternate sequences and scored them on a GPU to obtain scalar predictions, then converted them to log_2_ FC using the shared definition.

### ISM analysis of DREAM-RNN

We performed single-nucleotide ISM on the 110-bp inputs by iterating over each position p=1,…,110 and substituting the native nucleotide with each of the three alternatives (n∈{A,C,G,T}\{ref}); we then computed Δ(p,n), constructed the reference-average matrix, and visualized sequence logos.

### Massively parallel reporter assay (MPRA) evaluation of promoter variants

We evaluated Shorkie’s predictive performance for regulatory variants measured by a publicly available MPRA experiment^93^. The MPRA included five single-sequence sub-libraries: high_exp, low_exp, yeast_exp, random_exp, challenging_exp, each containing unique 110 bp promoter sequences, and three dual-sequence sub-libraries: SNVs_exp, motif_perturbation_exp, motif_tiling_exp, consisting of paired reference and alternate sequences. We excluded constructs present in any public leaderboard and randomly drew a uniform sample of 1,000 sequences without replacement from each category.

### Gene selection and library sampling

We stratified $T_{0}$ RNA-Seq expression into three quantiles (5–25%, 25–75%, 75–95%) separately by strand. From each quantile, we randomly chose three to four genes, resulting in 22 genes:

- Forward strand genes (10 total):
  - 5–25%: GPM3/SLI1/VPS52
  - 25–75%:YMR160W/MRPS28/YCT1
  - 75–95% RDL2/PHS1/RTC3/MSN4
- Reverse strand genes (12 total):
  - 5–25%: COA4/ERI1/RSM25/AIM11
  - 25–75% ERD1/MRM2/SNT2/MRPL1
  - 75–95% CSI2/RPE1/PKC1/MAE1

#### Promoter insertion design

To examine how Shorkie’s predictions vary relative to TSS, we first defined a promoter insertion window of 110 bp (±55 bp) and enforced a minimum 100-bp offset from the TSS to avoid direct overlap. We then chose eleven distinct insertion offsets (ranging from 100 to 200 bp, incremented by 10 bp). For each selected gene, we calculated the insertion midpoints as follows:

- Forward strand: midpoint = TSS − offset
- Reverse strand: midpoint = TSS + offset

Each midpoint defined a 110 bp replacement window from (midpoint − 55 bp) to (midpoint + 55 bp). We then replaced these native genomic windows with each MPRA-derived sequence and used Shorkie to predict the regulatory impact. In contrast, DREAM challenge models directly utilized the 110 bp MPRA windows as input to predict scalar expression scores.

#### Promoter variants effect quantification

To quantify gene-level effects from Shorkie’s predictions, we identified model-predicted coverage bins B(g) overlapping the exonic regions of each target gene (g).

For single-sequence libraries, we computed ${Cov}_{\text{native}}=\sum_{i\in ℬ(g)}y_{\text{native}}(i)$ and ${Cov}_{\text{MPRA}}=\sum_{i\in ℬ(g)}y_{\text{MPRA}}(i)$. We reported the regulatory effect as ${log}_{2}{FC}_{\text{single}}={log}_{2}\left({Cov}_{\text{MPRA}}+1\right)-{log}_{2}\left({Cov}_{\text{native}}+1\right)$. ${log}_{2}\;{FC}_{\text{single}}$ reflects changes in predicted coverage due to the MPRA insert relative to the native sequence.

In dual-sequence libraries, each construct includes both reference and alternate promoter variants. Shorkie predicted REF and ALT coverage separately, and we computed ${log}_{2}\;{FC}_{\text{ref}}$ and ${log}_{2}\;{FC}_{\text{alt}}$ relative to the native promoter, and summarized the predicted differential effect as $\Delta {log}_{2}\;{FC}_{\text{dual}}={log}_{2}\;{FC}_{\text{alt}}-{log}_{2}\;{FC}_{\text{ref}}$.

Experimental reporter assays yielded corresponding expression scores for the reference ($S_{\text{ref}}$) and alternate $\left(S_{\text{alt}}\right)$ sequences. We quantified their difference as $\Delta S=S_{\text{alt}}-S_{\text{ref}}$. Finally, we visualized scatterplots to assess the concordance between the Shorkie-predicted $\Delta {log}_{2}\;{FC}_{\text{dual}}$ and the experimentally derived ΔS scores.

## Supplementary Material

Supplementary Files

This is a list of supplementary files associated with this preprint. Click to download.

shorkiesupplementaltables.pdf

shorkiesupplemental gures.pdf

## Data Availability

The new Induction Dynamics Gene Expression Atlas RNA-seq datasets generated by Calico Life Sciences LLC are hosted on Google Cloud Storage (GCS). Coverage tracks (BigWig) are available at gs://shorkie-paper/data/supervised/bigwigs/, and processed TFRecords are at gs://shorkie-paper/data/supervised/processed. The genomes used for self-supervised language-model pretraining are at gs://shorkie-paper/data/unsupervised/genome/, with corresponding TFRecords at gs://shorkie-paper/data/unsupervised/processed/. The Shorkie LM and Shorkie models are implemented in TensorFlow. Shorkie LM is available at: gs://seqnn-share/shorkie_lm/. Shorkie models are available at: gs://seqnn-share/shorkie/. Parameters, training code and evaluation scripts for both the Shorkie LM and Shorkie models are available under the Apache-2.0 license at https://github.com/calico/baskerville-yeast and https://github.com/calico/shorkie-paper under license Apache-2.0.

## References

[1] Harbison C. T. Transcriptional regulatory code of a eukaryotic genome. Nature 431, 99–104 (2004). 15343339 10.1038/nature02800 PMC3006441

[2] He Q., Johnston J. & Zeitlinger J. ChIP-nexus enables improved detection of in vivo transcription factor binding footprints. Nat Biotechnol 33, 395–401 (2015). 25751057 10.1038/nbt.3121 PMC4390430

[3] Lee T. I. Transcriptional Regulatory Networks in *Saccharomyces cerevisiae*. Science 298, 799–804 (2002). 12399584 10.1126/science.1075090

[4] Rhee H. S. & Pugh B. F. Comprehensive Genome-wide Protein-DNA Interactions Detected at Single-Nucleotide Resolution. Cell 147, 1408–1419 (2011). 22153082 10.1016/j.cell.2011.11.013 PMC3243364

[5] Skene P. J. & Henikoff S. An efficient targeted nuclease strategy for high-resolution mapping of DNA binding sites. eLife 6, e21856 (2017). 28079019 10.7554/eLife.21856 PMC5310842

[6] Struhl K. Molecular mechanisms of transcriptional regulation in yeast. Annual review of biochemistry 58, 1051–1077 (1989). 10.1146/annurev.bi.58.070189.005155 2673007

[7] Venters B. J. A Comprehensive Genomic Binding Map of Gene and Chromatin Regulatory Proteins in Saccharomyces. Molecular Cell 41, 480–492 (2011). 21329885 10.1016/j.molcel.2011.01.015 PMC3057419

[8] Venters B. J. & Pugh B. F. A canonical promoter organization of the transcription machinery and its regulators in the *Saccharomyces* genome. Genome Res. 19, 360–371 (2009). 19124666 10.1101/gr.084970.108 PMC2661807

[9] Weiner A. High-Resolution Chromatin Dynamics during a Yeast Stress Response. Molecular Cell 58, 371–386 (2015). 25801168 10.1016/j.molcel.2015.02.002 PMC4405355

[10] Zentner G. E., Kasinathan S., Xin B., Rohs R. & Henikoff S. ChEC-seq kinetics discriminates transcription factor binding sites by DNA sequence and shape in vivo. Nat Commun 6, 8733 (2015). 26490019 10.1038/ncomms9733 PMC4618392

[11] Zhu J. Integrating large-scale functional genomic data to dissect the complexity of yeast regulatory networks. Nat Genet 40, 854–861 (2008). 18552845 10.1038/ng.167 PMC2573859

[12] Beer M. A. & Tavazoie S. Predicting Gene Expression from Sequence. Cell 117, 185–198 (2004). 15084257 10.1016/s0092-8674(04)00304-6

[13] Sharon E. Inferring gene regulatory logic from high-throughput measurements of thousands of systematically designed promoters. Nat Biotechnol 30, 521–530 (2012). 22609971 10.1038/nbt.2205 PMC3374032

[14] De Boer C. G. Deciphering eukaryotic gene-regulatory logic with 100 million random promoters. Nat Biotechnol 38, 56–65 (2020). 31792407 10.1038/s41587-019-0315-8 PMC6954276

[15] Vaishnav E. D. The evolution, evolvability and engineering of gene regulatory DNA. Nature 603, 455–463 (2022). 35264797 10.1038/s41586-022-04506-6 PMC8934302

[16] Zhou Z. DNABERT-2: Efficient Foundation Model and Benchmark For Multi-Species Genome. Preprint at 10.48550/ARXIV.2306.15006 (2023).

[17] Ji Y., Zhou Z., Liu H. & Davuluri R. V. DNABERT: pre-trained Bidirectional Encoder Representations from Transformers model for DNA-language in genome. Bioinformatics 37, 2112–2120 (2021). 33538820 10.1093/bioinformatics/btab083 PMC11025658

[18] Nguyen E. Sequence modeling and design from molecular to genome scale with Evo. Science 386, eado9336 (2024). 39541441 10.1126/science.ado9336 PMC12057570

[19] Zhai J. Cross-species modeling of plant genomes at single nucleotide resolution using a pre-trained DNA language model. Preprint at 10.1101/2024.06.04.596709 (2024). PMC12184517 40489624

[20] Benegas G., Batra S. S. & Song Y. S. DNA language models are powerful predictors of genome-wide variant effects. Proc. Natl. Acad. Sci. U.S.A. 120, e2311219120 (2023). 37883436 10.1073/pnas.2311219120 PMC10622914

[21] Sanabria M., Hirsch J., Joubert P. M. & Poetsch A. R. DNA language model GROVER learns sequence context in the human genome. Nat Mach Intell 6, 911–923 (2024).

[22] Karollus A. Species-aware DNA language models capture regulatory elements and their evolution. Genome Biol 25, 83 (2024). 38566111 10.1186/s13059-024-03221-x PMC10985990

[23] Dalla-Torre H. Nucleotide Transformer: building and evaluating robust foundation models for human genomics. Nat Methods 22, 287–297 (2025). 39609566 10.1038/s41592-024-02523-z PMC11810778

[24] Dao T. & Gu A. Transformers are SSMs: Generalized Models and Efficient Algorithms Through Structured State Space Duality. Preprint at 10.48550/ARXIV.2405.21060 (2024).

[25] Brixi G. Genome modeling and design across all domains of life with Evo 2. Preprint at 10.1101/2025.02.18.638918 (2025). PMC13128491 41781614

[26] Schiff Y. Caduceus: Bi-Directional Equivariant Long-Range DNA Sequence Modeling. Preprint at 10.48550/ARXIV.2403.03234 (2024). PMC12189541 40567809

[27] Nguyen E. HyenaDNA: Long-Range Genomic Sequence Modeling at Single Nucleotide Resolution. Preprint at 10.48550/ARXIV.2306.15794 (2023).

[28] Hayes T. Simulating 500 million years of evolution with a language model. Science 387, 850–858 (2025). 39818825 10.1126/science.ads0018

[29] Madani A. Large language models generate functional protein sequences across diverse families. Nat Biotechnol 41, 1099–1106 (2023). 36702895 10.1038/s41587-022-01618-2 PMC10400306

[30] Nijkamp E., Ruffolo J. A., Weinstein E. N., Naik N. & Madani A. ProGen2: Exploring the boundaries of protein language models. Cell Systems 14, 968–978.e3 (2023). 37909046 10.1016/j.cels.2023.10.002

[31] Bhatnagar A. Scaling unlocks broader generation and deeper functional understanding of proteins. Preprint at 10.1101/2025.04.15.649055 (2025).

[32] Lin Z. Evolutionary-scale prediction of atomic-level protein structure with a language model. Science 379, 1123–1130 (2023). 36927031 10.1126/science.ade2574

[33] Shen X.-X. Tempo and Mode of Genome Evolution in the Budding Yeast Subphylum. Cell 175, 1533–1545.e20 (2018). 30415838 10.1016/j.cell.2018.10.023 PMC6291210

[34] Hackett S. R. Learning causal networks using inducible transcription factors and transcriptome-wide time series. Molecular Systems Biology 16, e9174 (2020). 32181581 10.15252/msb.20199174 PMC7076914

[35] Avsec Ž. Effective gene expression prediction from sequence by integrating long-range interactions. Nat Methods 18, 1196–1203 (2021). 34608324 10.1038/s41592-021-01252-x PMC8490152

[36] Linder J., Srivastava D., Yuan H., Agarwal V. & Kelley D. R. Predicting RNA-seq coverage from DNA sequence as a unifying model of gene regulation. Nat Genet (2025) doi:10.1038/s41588-024-02053-6. PMC11985352 39779956

[37] Ronneberger O., Fischer P. & Brox T. U-Net: Convolutional Networks for Biomedical Image Segmentation. in Medical Image Computing and Computer-Assisted Intervention – MICCAI 2015 (eds. Navab N., Hornegger J., Wells W. M. & Frangi A. F.) vol. 9351 234–241 (Springer International Publishing, Cham, 2015).

[38] Huerta-Cepas J., Serra F. & Bork P. ETE 3: Reconstruction, Analysis, and Visualization of Phylogenomic Data. Mol Biol Evol 33, 1635–1638 (2016). 26921390 10.1093/molbev/msw046 PMC4868116

[39] Letunic I. & Bork P. Interactive Tree of Life (iTOL) v6: recent updates to the phylogenetic tree display and annotation tool. Nucleic Acids Research 52, W78–W82 (2024). 38613393 10.1093/nar/gkae268 PMC11223838

[40] Letunic I. & Bork P. Interactive Tree Of Life (iTOL): an online tool for phylogenetic tree display and annotation. Bioinformatics 23, 127–128 (2007). 17050570 10.1093/bioinformatics/btl529

[41] Marçais G. MUMmer4: A fast and versatile genome alignment system. PLoS Comput Biol 14, e1005944 (2018). 29373581 10.1371/journal.pcbi.1005944 PMC5802927

[42] Ondov B. D. Mash: fast genome and metagenome distance estimation using MinHash. Genome Biol 17, 132 (2016). 27323842 10.1186/s13059-016-0997-x PMC4915045

[43] Bailey T. L. MEME SUITE: tools for motif discovery and searching. Nucleic Acids Research 37, W202–W208 (2009). 19458158 10.1093/nar/gkp335 PMC2703892

[44] Flynn J. M. RepeatModeler2 for automated genomic discovery of transposable element families. Proc. Natl. Acad. Sci. U.S.A. 117, 9451–9457 (2020). 32300014 10.1073/pnas.1921046117 PMC7196820

[45] Smit A. F. A., Hubley R. & Green P. RepeatMasker Open-4.0. (2013).

[46] Storer J., Hubley R., Rosen J., Wheeler T. J. & Smit A. F. The Dfam community resource of transposable element families, sequence models, and genome annotations. Mobile DNA 12, 2 (2021). 33436076 10.1186/s13100-020-00230-y PMC7805219

[47] Ou S. & Jiang N. LTR_retriever: A Highly Accurate and Sensitive Program for Identification of Long Terminal Repeat Retrotransposons. Plant Physiol. 176, 1410–1422 (2018). 29233850 10.1104/pp.17.01310 PMC5813529

[48] Price A. L., Jones N. C. & Pevzner P. A. De novo identification of repeat families in large genomes. Bioinformatics 21, i351–i358 (2005). 15961478 10.1093/bioinformatics/bti1018

[49] Li H. Minimap2: pairwise alignment for nucleotide sequences. Bioinformatics 34, 3094–3100 (2018). 29750242 10.1093/bioinformatics/bty191 PMC6137996

[50] Rafi A. M., Kiyota B., Yachie N. & De Boer C. Detecting and avoiding homology-based data leakage in genome-trained sequence models. Preprint at 10.1101/2025.01.22.634321 (2025).

[51] Sahu B. Sequence determinants of human gene regulatory elements. Nat Genet 54, 283–294 (2022). 35190730 10.1038/s41588-021-01009-4 PMC8920891

[52] Tomaz Da Silva P. Nucleotide dependency analysis of DNA language models reveals genomic functional elements. Preprint at 10.1101/2024.07.27.605418 (2024). PMC12513839 41073788

[53] Shrikumar A. Technical Note on Transcription Factor Motif Discovery from Importance Scores (TF-MoDISco) version 0.5.6.5. Preprint at 10.48550/ARXIV.1811.00416 (2018).

[54] Shrikumar A. Technical Note on Transcription Factor Motif Discovery from Importance Scores (TF-MoDISco) version 0.5.6.5. Preprint at 10.48550/arXiv.1811.00416 (2020).

[55] De Boer C. G. & Hughes T. R. YeTFaSCo: a database of evaluated yeast transcription factor sequence specificities. Nucleic Acids Research 40, D169–D179 (2012). 22102575 10.1093/nar/gkr993 PMC3245003

[56] MacIsaac K. D. An improved map of conserved regulatory sites for Saccharomyces cerevisiae. BMC Bioinformatics 7, (2006). 10.1186/1471-2105-7-113 PMC1435934 16522208

[57] Newburger D. E. & Bulyk M. L. UniPROBE: an online database of protein binding microarray data on protein-DNA interactions. Nucleic Acids Research 37, D77–D82 (2009). 18842628 10.1093/nar/gkn660 PMC2686578

[58] Pachkov M., Erb I., Molina N. & Van Nimwegen E. SwissRegulon: a database of genome-wide annotations of regulatory sites. Nucleic Acids Research 35, D127–D131 (2007). 17130146 10.1093/nar/gkl857 PMC1716717

[59] Teixeira M. C. The YEASTRACT database: an upgraded information system for the analysis of gene and genomic transcription regulation in *Saccharomyces cerevisiae*. Nucl. Acids Res. 42, D161–D166 (2014). 24170807 10.1093/nar/gkt1015 PMC3965121

[60] Zhu J. & Zhang M. Q. SCPD: a promoter database of the yeast Saccharomyces cerevisiae. Bioinformatics 15, 607–611 (1999). 10487868 10.1093/bioinformatics/15.7.607

[61] Yabana N. & Yamamoto M. *Schizosaccharomyces pombe map1*^+^ Encodes a MADS-Box-Family Protein Required for Cell-Type-Specific Gene Expression. Molecular and Cellular Biology 16, 3420–3428 (1996). 8668157 10.1128/mcb.16.7.3420 PMC231336

[62] Nielsen O., Friis T. & Kjærulff S. The Schizosaccharomyces pombe map1 gene encodes an SRF / MCM1-related protein required for P-cell specific gene expression. Mol Gen Genet 253, 387–392 (1996). 9003326 10.1007/pl00008604

[63] Casselton L. A. Mate recognition in fungi. Heredity 88, 142–147 (2002). 11932772 10.1038/sj.hdy.6800035

[64] Oliva A. The Cell Cycle–Regulated Genes of Schizosaccharomyces pombe. PLoS Biol 3, e225 (2005). 15966770 10.1371/journal.pbio.0030225 PMC1157095

[65] Rossi M. J. A high-resolution protein architecture of the budding yeast genome. Nature 592, 309–314 (2021). 33692541 10.1038/s41586-021-03314-8 PMC8035251

[66] Caudal É. Pan-transcriptome reveals a large accessory genome contribution to gene expression variation in yeast. Nat Genet 56, 1278–1287 (2024). 38778243 10.1038/s41588-024-01769-9 PMC11176082

[67] Martin D. E., Soulard A. & Hall M. N. TOR Regulates Ribosomal Protein Gene Expression via PKA and the Forkhead Transcription Factor FHL1. Cell 119, 969–979 (2004). 15620355 10.1016/j.cell.2004.11.047

[68] Schawalder S. B. Growth-regulated recruitment of the essential yeast ribosomal protein gene activator Ifh1. Nature 432, 1058–1061 (2004). 15616569 10.1038/nature03200

[69] Rudra D., Zhao Y. & Warner J. R. Central role of Ifh1p–Fhl1p interaction in the synthesis of yeast ribosomal proteins. EMBO J 24, 533–542 (2005). 15692568 10.1038/sj.emboj.7600553 PMC548658

[70] Reja R., Vinayachandran V., Ghosh S. & Pugh B. F. Molecular mechanisms of ribosomal protein gene coregulation. Genes Dev. 29, 1942–1954 (2015). 26385964 10.1101/gad.268896.115 PMC4579351

[71] Shore D. RAP1: a protean regulator in yeast. Trends in Genetics 10, 408–412 (1994). 7809947 10.1016/0168-9525(94)90058-2

[72] Wade J. T., Hall D. B. & Struhl K. The transcription factor Ifh1 is a key regulator of yeast ribosomal protein genes. Nature 432, 1054–1058 (2004). 15616568 10.1038/nature03175

[73] Warner J. R. The economics of ribosome biosynthesis in yeast. Trends in Biochemical Sciences 24, 437–440 (1999). 10542411 10.1016/s0968-0004(99)01460-7

[74] Jorgensen P. A dynamic transcriptional network communicates growth potential to ribosome synthesis and critical cell size. Genes Dev. 18, 2491–2505 (2004). 15466158 10.1101/gad.1228804 PMC529537

[75] Wade C. H., Umbarger M. A. & McAlear M. A. The budding yeast rRNA and ribosome biosynthesis (RRB) regulon contains over 200 genes. Yeast 23, 293–306 (2006). 16544271 10.1002/yea.1353

[76] Arnone J. T. & McAlear M. A. Adjacent Gene Pairing Plays a Role in the Coordinated Expression of Ribosome Biogenesis Genes *MPP10* and *YJR003C* in Saccharomyces cerevisiae. Eukaryot Cell 10, 43–53 (2011). 21115740 10.1128/EC.00257-10 PMC3019797

[77] Hughes J. D., Estep P. W., Tavazoie S. & Church G. M. Computational identification of Cis -regulatory elements associated with groups of functionally related genes in Saccharomyces cerevisiae 1 1Edited by F. E. Cohen. Journal of Molecular Biology 296, 1205–1214 (2000). 10698627 10.1006/jmbi.2000.3519

[78] Jorgensen P., Nishikawa J. L., Breitkreutz B.-J. & Tyers M. Systematic Identification of Pathways That Couple Cell Growth and Division in Yeast. Science 297, 395–400 (2002). 12089449 10.1126/science.1070850

[79] Brown S. J., Cole M. D. & Erives A. J. Evolution of the holozoan ribosome biogenesis regulon. BMC Genomics 9, 442 (2008). 18816399 10.1186/1471-2164-9-442 PMC2570694

[80] Robinson J. T., Thorvaldsdottir H., Turner D. & Mesirov J. P. igv.js: an embeddable JavaScript implementation of the Integrative Genomics Viewer (IGV). Bioinformatics 39, btac830 (2023). 36562559 10.1093/bioinformatics/btac830 PMC9825295

[81] Schirman D., Yakhini Z., Pilpel Y. & Dahan O. A broad analysis of splicing regulation in yeast using a large library of synthetic introns. PLoS Genet 17, e1009805 (2021). 34570750 10.1371/journal.pgen.1009805 PMC8496845

[82] Moore M. J., Query C. C., Sharp P. A., & others. Splicing of precursors to mRNAs by the spliceosome. Cold Spring Harbor Monograph Series 24, 303–303 (1993).

[83] Parker R., Siliciano P. G. & Guthrie C. Recognition of the TACTAAC box during mRNA splicing in yeast involves base pairing to the U2-like snRNA. Cell 49, 229–239 (1987). 3552247 10.1016/0092-8674(87)90564-2

[84] Zavanelli M. I. & Ares M. Efficient association of U2 snRNPs with pre-mRNA requires an essential U2 RNA structural element. Genes & Development 5, 2521–2533 (1991). 1752442 10.1101/gad.5.12b.2521

[85] Engel S. R. *Saccharomyces* Genome Database: advances in genome annotation, expanded biochemical pathways, and other key enhancements. GENETICS 229, iyae185 (2025). 39530598 10.1093/genetics/iyae185 PMC11912841

[86] Garmendia-Torres C., Goldbeter A. & Jacquet M. Nucleocytoplasmic Oscillations of the Yeast Transcription Factor Msn2: Evidence for Periodic PKA Activation. Current Biology 17, 1044–1049 (2007). 17570669 10.1016/j.cub.2007.05.032

[87] Ni L. Dynamic and complex transcription factor binding during an inducible response in yeast. Genes Dev. 23, 1351–1363 (2009). 19487574 10.1101/gad.1781909 PMC2701586

[88] Martínez-Pastor M. T. The Saccharomyces cerevisiae zinc finger proteins Msn2p and Msn4p are required for transcriptional induction through the stress response element (STRE). The EMBO Journal 15, 2227–2235 (1996). 8641288 PMC450147

[89] Gasch A. P. Genomic Expression Programs in the Response of Yeast Cells to Environmental Changes. MBoC 11, 4241–4257 (2000). 11102521 10.1091/mbc.11.12.4241 PMC15070

[90] Causton H. C. Remodeling of Yeast Genome Expression in Response to Environmental Changes. MBoC 12, 323–337 (2001). 11179418 10.1091/mbc.12.2.323 PMC30946

[91] Kuras L., Barbey R. & Thomas D. Assembly of a bZIP-bHLH transcription activation complex: formation of the yeast Cbf1-Met4-Met28 complex is regulated through Met28 stimulation of Cbf1 DNA binding. The EMBO Journal 16, 2441–2451 (1997). 9171357 10.1093/emboj/16.9.2441 PMC1169844

[92] Lee T. A. Dissection of Combinatorial Control by the Met4 Transcriptional Complex. MBoC 21, 456–469 (2010). 19940020 10.1091/mbc.E09-05-0420 PMC2814790

[93] Rafi A. M. A community effort to optimize sequence-based deep learning models of gene regulation. Nat Biotechnol (2024) doi:10.1038/s41587-024-02414-w. PMC12339383 39394483

[94] Kita R., Venkataram S., Zhou Y. & Fraser H. B. High-resolution mapping of *cis* -regulatory variation in budding yeast. Proc. Natl. Acad. Sci. U.S.A. 114, (2017). 10.1073/pnas.1717421114 PMC5740631 29183975

[95] Peter J. Genome evolution across 1,011 Saccharomyces cerevisiae isolates. Nature 556, 339–344 (2018). 29643504 10.1038/s41586-018-0030-5 PMC6784862

[96] Kuras L. Dual Regulation of the Met4 Transcription Factor by Ubiquitin-Dependent Degradation and Inhibition of Promoter Recruitment. Molecular Cell 10, 69–80 (2002). 12150908 10.1016/s1097-2765(02)00561-0

[97] Leroy C., Cormier L. & Kuras L. Independent Recruitment of Mediator and SAGA by the Activator Met4. Molecular and Cellular Biology 26, 3149–3163 (2006). 16581789 10.1128/MCB.26.8.3149-3163.2006 PMC1446967

[98] Lin L., Chamberlain L., Zhu L. J. & Green M. R. Analysis of Gal4-directed transcription activation using Tra1 mutants selectively defective for interaction with Gal4. Proc. Natl. Acad. Sci. U.S.A. 109, 1997–2002 (2012). 22308403 10.1073/pnas.1116340109 PMC3277556

[99] Chandrasekaran S. & Skowyra D. The emerging regulatory potential of SCFMet30 -mediated polyubiquitination and proteolysis of the Met4 transcriptional activator. Cell Div 3, 11 (2008). 18655704 10.1186/1747-1028-3-11 PMC2526995

[100] Hendrycks D. & Gimpel K. Gaussian Error Linear Units (GELUs). Preprint at 10.48550/ARXIV.1606.08415 (2016).

[101] Kurtz S. Versatile and open software for comparing large genomes. Genome Biol 5, R12 (2004). 14759262 10.1186/gb-2004-5-2-r12 PMC395750

[102] Baker D. N. & Langmead B. Genomic sketching with multiplicities and locality-sensitive hashing using Dashing 2. Genome Res. gr.277655.123 (2023) doi:10.1101/gr.277655.123. PMC10538361 37414575

[103] Ertl O. SetSketch: Filling the Gap between MinHash and HyperLogLog. (2021) doi:10.48550/ARXIV.2101.00314.

[104] Ertl O. ProbMinHash – A Class of Locality-Sensitive Hash Algorithms for the (Probability) Jaccard Similarity. IEEE Trans. Knowl. Data Eng. 1–1 (2020) doi:10.1109/TKDE.2020.3021176.

[105] Broder A. Z. On the resemblance and containment of documents. in Proceedings. Compression and Complexity of SEQUENCES 1997 (Cat. No.97TB100171) 21–29 (IEEE Comput. Soc, Salerno, Italy, 1998). doi:10.1109/SEQUEN.1997.666900.

[106] Jaccard P. Étude comparative de la distribution florale dans une portion des Alpes et des Jura. Bull Soc Vaudoise Sci Nat 37, 547–579 (1901).

[107] Wheeler T. J. Dfam: a database of repetitive DNA based on profile hidden Markov models. Nucleic Acids Research 41, D70–D82 (2012). 23203985 10.1093/nar/gks1265 PMC3531169

[108] Pertea G. & Pertea M. GFF Utilities: GffRead and GffCompare. F1000Res 9, 304 (2020). 10.12688/f1000research.23297.1 PMC7222033 32489650

[109] Simão F. A., Waterhouse R. M., Ioannidis P., Kriventseva E. V. & Zdobnov E. M. BUSCO: assessing genome assembly and annotation completeness with single-copy orthologs. Bioinformatics 31, 3210–3212 (2015). 26059717 10.1093/bioinformatics/btv351

[110] Devlin J., Chang M.-W., Lee K. & Toutanova K. BERT: Pre-training of Deep Bidirectional Transformers for Language Understanding. Preprint at 10.48550/ARXIV.1810.04805 (2018).

[111] Mallet V. & Vert J.-P. Reverse-complement equivariant networks for DNA sequences. Advances in neural information processing systems 34, 13511–13523 (2021).

[112] Zhou H., Shrikumar A. & Kundaje A. Towards a better understanding of reverse-complement equivariance for deep learning models in genomics. in Machine Learning in Computational Biology 1–33 (PMLR, 2022).

[113] Kingma D. P. & Ba J. Adam: A Method for Stochastic Optimization. Preprint at 10.48550/ARXIV.1412.6980 (2014).

[114] Schneider T. D., Stormo G. D., Gold L. & Ehrenfeucht A. Information content of binding sites on nucleotide sequences. Journal of Molecular Biology 188, 415–431 (1986). 3525846 10.1016/0022-2836(86)90165-8

[115] Schneider T. D. & Stephens R. M. Sequence logos: a new way to display consensus sequences. Nucl Acids Res 18, 6097–6100 (1990). 2172928 10.1093/nar/18.20.6097 PMC332411

[116] Wolf T. HuggingFace’s Transformers: State-of-the-art Natural Language Processing. Preprint at 10.48550/ARXIV.1910.03771 (2019).

[117] Hinton G. E. & Roweis S. Stochastic neighbor embedding. Advances in neural information processing systems 15, (2002).

[118] Arita Y. A genome-scale yeast library with inducible expression of individual genes. Molecular Systems Biology 17, e10207 (2021). 34096681 10.15252/msb.202110207 PMC8182650

[119] Balakrishnan R. YeastMine—an integrated data warehouse for Saccharomyces cerevisiae data as a multipurpose tool-kit. Database 2012, (2012). 10.1093/database/bar062 PMC3308152 22434830

[120] Turco G. Global analysis of the yeast knockout phenome. Sci. Adv. 9, eadg5702 (2023). 37235661 10.1126/sciadv.adg5702 PMC11326039

[121] Hou J. The Hidden Complexity of Mendelian Traits across Natural Yeast Populations. Cell Reports 16, 1106–1114 (2016). 27396326 10.1016/j.celrep.2016.06.048 PMC4963288

[122] Li H. Aligning sequence reads, clone sequences and assembly contigs with BWA-MEM. Preprint at 10.48550/ARXIV.1303.3997 (2013).

[123] Li H. The Sequence Alignment/Map format and SAMtools. Bioinformatics 25, 2078–2079 (2009). 19505943 10.1093/bioinformatics/btp352 PMC2723002

[124] Quinlan A. R. & Hall I. M. BEDTools: a flexible suite of utilities for comparing genomic features. Bioinformatics 26, 841–842 (2010). 20110278 10.1093/bioinformatics/btq033 PMC2832824

[125] Dobin A. STAR: ultrafast universal RNA-seq aligner. Bioinformatics 29, 15–21 (2013). 23104886 10.1093/bioinformatics/bts635 PMC3530905

[126] Picard toolkit. Broad Institute, GitHub repository (2019).

[127] Stovner E. B. & Sætrom P. PyRanges: efficient comparison of genomic intervals in Python. Bioinformatics 36, 918–919 (2020). 31373614 10.1093/bioinformatics/btz615

